# Saline-Alkali Soil Property Improved by the Synergistic Effects of *Priestia aryabhattai* JL-5, *Staphylococcus pseudoxylosus* XW-4, *Leymus chinensis* and Soil Microbiota

**DOI:** 10.3390/ijms24097737

**Published:** 2023-04-23

**Authors:** Yujue Wang, Yan Wang, Qian Zhang, Hangzhe Fan, Xinyu Wang, Jianan Wang, Ying Zhou, Zhanyu Chen, Fengjie Sun, Xiyan Cui

**Affiliations:** 1 College of Life Sciences, Jilin Agricultural University, Changchun 130118, Chinawangyansk@jlau.edu.cn (Y.W.);; 2 College of Agronomy, Jilin Agricultural University, Changchun 130118, China; 3 Department of Biological Sciences, School of Science and Technology, Georgia Gwinnett College, Lawrenceville, GA 30043, USA

**Keywords:** saline-alkali soil, *Priestia aryabhattai*, *Staphylococcus pseudoxylosus*, *Leymus chinensis*, saline-alkali resistance, soil microbiota

## Abstract

Two saline-alkali-tolerant bacterial strains, *Priestia aryabhattai* JL-5 and *Staphylococcus pseudoxylosus* XW-4, were isolated, with high capabilities of hydrolyzing phosphate and producing cellulase, respectively. The molecular mechanisms regulating the saline-alkali tolerance in the strain JL-5 were further investigated using transcriptome analysis. The contents of lactic acid and proline and the enzymatic activity of glutamine synthetase in the strain JL-5 were significantly increased. The properties of saline-alkali soils were significantly improved by the enhanced growth of the indicator plant *Leymus chinensis* under the combined applications of the strains JL-5 and XW-4 mixed with corn straw. The contents of catalase, peroxidase, superoxide dismutase and proline of *L. chinensis* were significantly increased, and the content of malondialdehyde was significantly decreased in the combined treatment of both bacterial strains. The contents of available nitrogen, phosphorus and potassium and organic matters in the soil treated with both strains were significantly increased, as well as the diversity and abundance of the soil microbiota. Our study evidently demonstrated the synergistic effects of the strains JL-5 and XW-4, indicator plants and the local microbiota in terms of improving the saline-alkali soil properties, providing strong experimental evidence to support the commercial development of the combined application of both strains to improve the properties of saline-alkali soils.

## 1. Introduction

China has the largest area of saline-alkali land worldwide [1]. The large amount of salt and alkali maintained in saline-alkali soils causes deleterious effects on the environments and organisms, e.g., disrupting the ecological balance between soil and water and changing the structure and properties of soil by affecting soil pore distribution, ultimately weakening the soil permeability and hardening the soil [2]. Furthermore, the declined fertility in the saline-alkali soils could harm the normal growth and yields of crops [3]. To date, the soil salinization has become one of the severe problems in agriculture worldwide, and solutions are urgently needed.

At present, the improvement of the saline-alkali soil property is mainly achieved by physical, chemical and bioremediation strategies [4]. The physical improvements of saline-alkali soil mainly include land leveling, soil improvement in micro areas, soil loosening, alternate wet and dry cultivation and soil surface coverage [5,6]. The chemical improvements refer to the transformation, adsorption or fixation of saline-alkali components in the soil through the application of chemical modifiers and organic matter [7,8] to reduce the contents of saline-alkali components in the soil. For example, the cation exchange capacity of saline-alkali soil is improved by using corn straw biochar to increase the contents of organic matters and nutrients in the saline-alkali soils [9]. Bioremediation improves the saline-alkali soils by applying microorganisms in soil and planting saline-alkali-tolerant plants [10]. It is expected that the combined applications of physical, chemical and bioremediation methods would achieve enhanced improvements of the saline-alkali soils.

The synergistic relationships are commonly known among the taxa in the soil microbiota and between microorganisms and plants, i.e., properties of saline-alkali soil could be improved by saline-alkali-tolerant plants, which in turn affect the microbial community in the soil, ultimately playing a synergistic role in the improvement of saline-alkali soil [11]. Furthermore, with the economic, environmental and ecological benefits integrated, the microbial remediation has played increasingly important functions in saline-alkali soil improvement, showing significant potential in the development of composite biological agents from microbes [12]. Similarly, the inoculation of microorganisms not only affects plants but also alters the taxonomic components of the local microbiota [13]. These studies have evidently demonstrated the synergistic effects among the microorganisms and plants as well as the local microbiota, showing significant potential in the bioremediation and improvements of various types of adverse soil environments.

Studies have identified many saline-alkali-tolerant bacterial and fungal taxa in the natural saline-alkali environments [14], and these native microorganisms are most suitable for growth in environments similar to those where they are originally isolated from. For example, the arbuscular mycorrhiza derived from the native soil has been used to promote the antioxidant enzyme system and the saline-alkali tolerance of wheat [15]. Furthermore, in order to survive and thrive, the native microorganisms in the saline-alkali environments would develop adaptive strategies to cope with the stressed environments [16], e.g., using a primary proton pump to capture or retain protons and producing acidic metabolites through carbohydrate and amino acid metabolisms [17,18]. Moreover, the increased salinity level in the saline-alkali soils could alter the microbial community structure and functional adaptability, such as enhanced carbohydrate and amino acid metabolisms, to cope with the high salt stress [19]. In recent years, with the development and popularization of next-generation DNA sequencing technology, more advanced methodologies have been established at the genomic and transcriptomic levels to investigate the adaptation mechanisms of organisms under the stress of external adverse factors [20,21,22].

Studies have shown that the interaction networks among various organisms are maintained at a dynamic status [23], i.e., a series of physiological and biochemical variations are detected in the microorganisms in response to the external stresses, while in turn, these variations could naturally affect other microorganisms due to the extremely large number of taxa in the soil microbiota [24] as well as the local plants [25], ultimately altering the original status of their synergistic relationships. Furthermore, studies have shown that the application of exogenous γ-aminobutyric acid (GABA) promotes the growth of nitrogen fixing rhizobia under the saline-alkali stress and enhances the saline-alkali tolerance and the symbiotic system of the local plant *Astragalus membranaceus*, providing an effective bioremediation strategy for improving the saline-alkali soil [26]. Moreover, as a gramineous plant native to China, Korea, Mongolia and Russia, *Leymus chinensis* is an indicator and dominant species with a large ecological amplitude in the saline-alkali soils in Northeastern China, playing an important role in improving the saline-alkali soils and indicating the degree of soil salinization [27]. In particular, the level of soil salinization could be represented by the oxidative stress response in *L. chinensis* caused by the changes in the contents of catalase (CAT), peroxidase (POD), superoxide dismutase (SOD), malondialdehyde (MAD) and proline under the saline-alkali stress [28,29].

In this study, two strains of saline-alkali-tolerant bacteria were isolated from saline-alkali soil in Northeastern China and identified as *Priestia aryabhattai* JL-5 and *Staphylococcus pseudoxylosus* XW-4, with high capabilities of hydrolyzing phosphate and producing cellulase, respectively, based on 16S rRNA and bacterial functional analyses. The goals of our study were to: (1) explore the molecular mechanisms underlying the saline-alkali resistance in the strain JL-5 using transcriptome analysis; (2) investigate the alleviation effects of the strains JL-5 and XW-4 on the saline-alkali soils based on the chemical and physiological properties of saline-alkali soils mixed with corn straw, which was used as the substrate of cellulose-degrading bacteria, including strain XW-4, to generate biochar and then treated with strain JL-5, strain XW-4 and a combination of both strains, respectively; and (3) provide experimental evidence to verify the synergistic effects among the strains JL-5 and XW-4, indicator plants of *L. chinensis* and taxa of local microbiota on the improvement of the saline-alkali soil property. Our study provided strong experimental evidence to support the further commercial development of the combined application of the strains JL-5 and XW-4 to improve the saline-alkali soil properties.

## 2. Results

### 2.1. Isolation and Identification of Saline-alkali-Tolerant Bacteria

**Figure 1:**
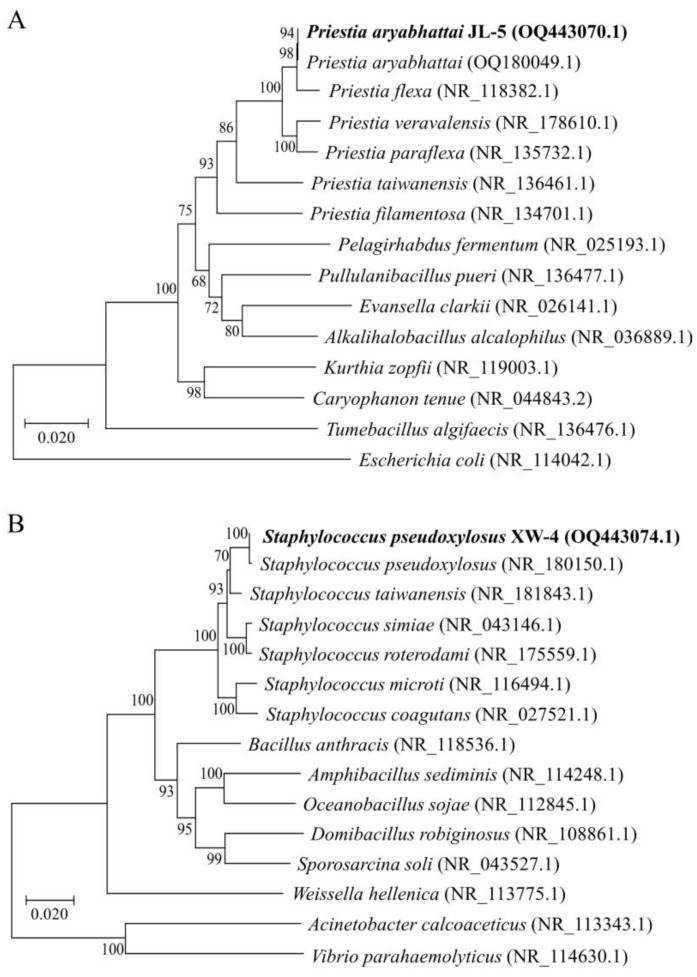
Identifications of (**A**) *Priestia aryabhattai* JL-5 and (**B**) *Staphylococcus pseudoxylosus* XW-4 based on phylogenetic analysis, i.e., the neighbor-joining trees constructed using 16S rRNA sequences, with *Escherichia coli* and *Acinetobacter calcoaceticus/Vibrio parahaemolyticus* used as the outgroups, respectively. The two bacterial taxa with 16S rRNA sequenced in this study are highlighted in bold. The GenBank accessions are given in the parentheses next to the names of the bacterial taxa. Bootstrap supports (%) based on 1000 replicates are given next to the branches on the tree.

**Figure 2:**
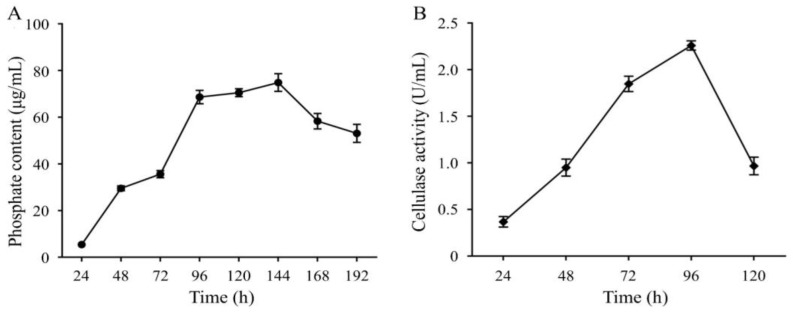
Phosphate-solubilizing ability of *Priestia aryabhattai* JL-5 (**A**) and cellulase-producing capability of *Staphylococcus pseudoxylosus* XW-4 (**B**). The error bars represent the standard deviations (n = 3) on each data point.

Two bacterial strains with high saline-alkali resistance and optimal growth under pH levels 7–10 were obtained by the isolation and functional screening of microorganisms in saline-alkali soil samples. Based on both comparative and phylogenetic analyses, these two strains were identified as *Priestia aryabhattai* JL-5 and *Staphylococcus pseudoxylosus* XW-4, respectively (Figure 1), showing high capabilities of dissolving phosphate and producing cellulase, respectively (Figure 2). The results showed that the phosphate-solubilizing capacity of strain JL-5 was first increased up to 6 d, reaching the highest levels from day 4 to day 6, and then decreased in the next 2 d. Similarly, the cellulose-degrading capability of the strain XW-4 was first increased from day 1, reaching the highest level (i.e., the strongest cellulolytic ability) in 4 d, and then decreased on day 5.

### 2.2. Transcriptome Sequencing and Data Analysis

#### 2.2.1. Selection of High-Quality Transcriptome Sequences

**Figure 3:**
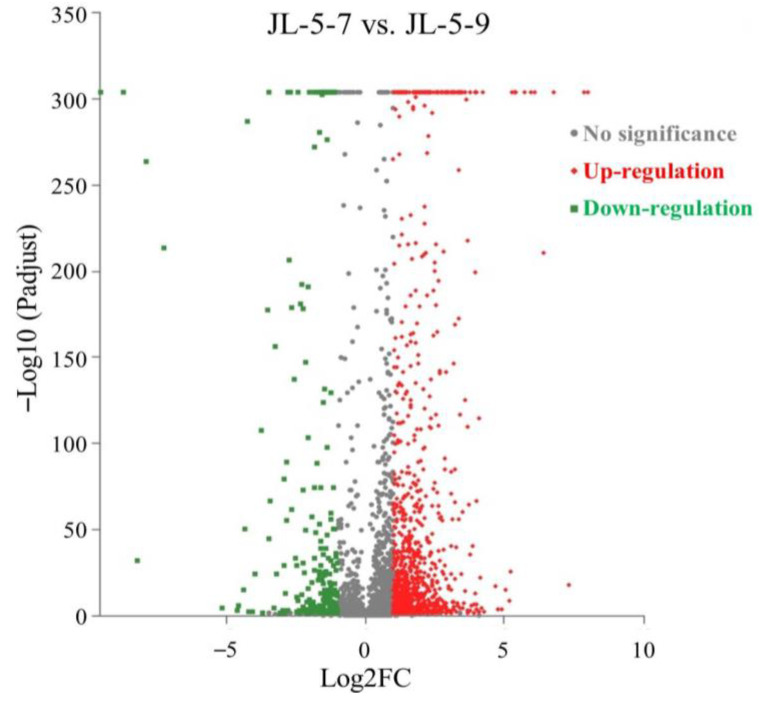
Volcano plot of a total of 1,304 differentially expressed genes (1066 up-regulated and 238 down-regulated) identified in *Priestia aryabhattai* JL-5 under saline-alkali stress (JL-5-9) compared with the control (JL-5-7).

In order to identify the genes involved in the molecular response of the strain JL-5 to the saline-alkali stress, transcriptome sequencing was performed using the strain JL-5 cultured at pH 7.0 and 1% salt (i.e., the control labeled as strain JL-5-7) and pH 9.0 and 2% salt (i.e., the saline-alkali condition labeled as strain JL-5-9), respectively. The transcriptome sequencing and assembly characteristics are summarized in Table 1. The results showed that a total of 30,721,042 and 29,035,034 raw reads were generated under the control and saline-alkali conditions, with 28,333,104 (92.2%) and 26,955,132 (92.8%) reads mapped to the reference genome of *Bacillus cereus* (GenBank accession GCF_002220285.1 with genome assembly ASM222028v1). In comparison with the control, a total of 1304 differentially expressed genes (DEGs; 1,066 up-regulated and 238 down-regulated) were identified in the strain JL-5 under the saline-alkali condition (Figure 3; Table S1).

#### 2.2.2. GO Annotation and KEGG Enrichment Analyses of Differentially Expressed Genes

**Figure 4:**
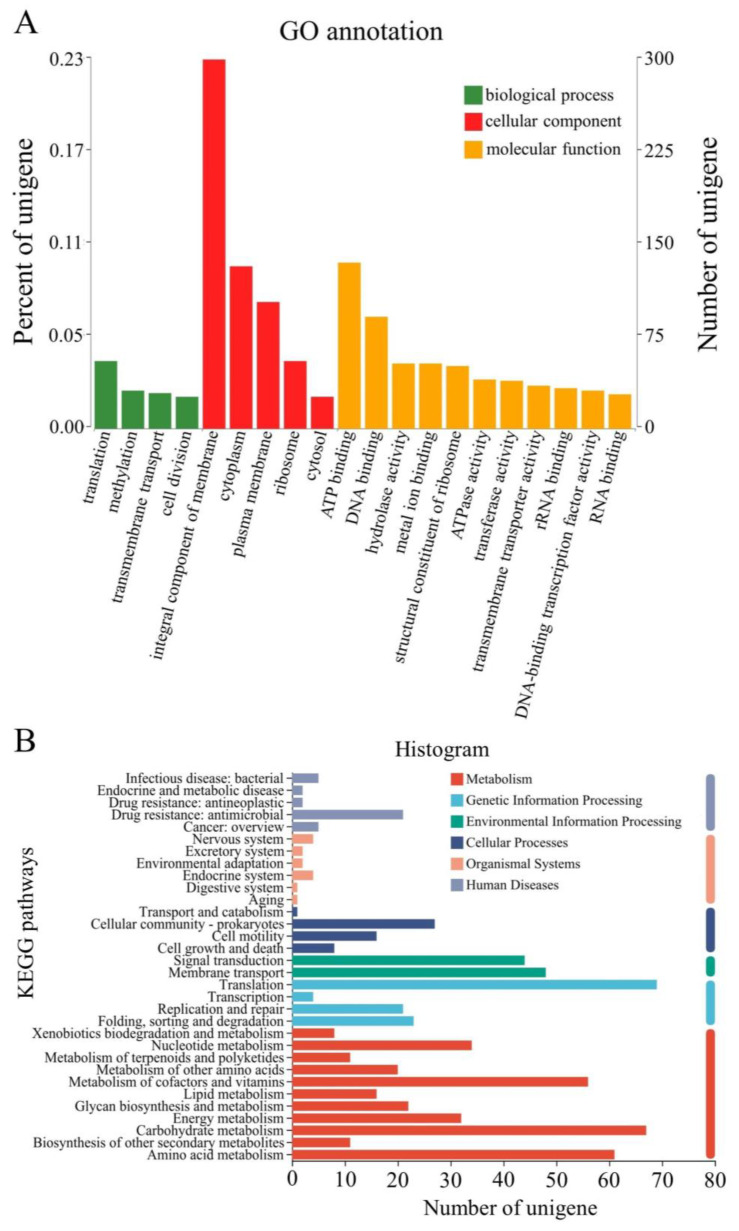
GO annotation (**A**) and KEGG enrichment analysis (**B**) of differentially expressed genes (DEGs) identified in *Priestia aryabhattai* JL-5 under the saline-alkali conditions.

The functions of the DEGs identified in the strain JL-5 under the saline-alkali stress were annotated by the Gene Ontology (GO) database into three categories of GO terms, i.e., biological process, cellular component and molecular function, with the highest number of genes annotated in translation, the integral component of the membrane and ATP binding, respectively (Figure 4A). Further examinations of the annotated genes revealed that the functions of these genes were involved in: (1) translation and cellular components, including macromolecular substances, peptides, amides, organics and nitrogen compounds; (2) cell metabolisms related to protein, nitrogen, peptide and organic nitrate; (3) iron sulfur cluster assembly, ribosome composition and biosynthesis; and (4) deoxyribose packaging, cell component assembly and non-membrane-bound organelle function. In particular, the genes related to the synthesis of cell components were particularly active, e.g., the negative regulatory gene *SpoVG* involved in cell septation regulation was significantly down-regulated. The expressions of genes regulating nitrogen metabolism and nitrogen-containing compounds and nitrates in cellular and non-cellular components were also significantly altered by the saline-alkali stress. The expressions of genes involved in peptide, protein and amide biosynthesis were also altered by saline-alkali stress, e.g., the gene *glnA* involved in glutamine synthesis was the most significantly up-regulated.

The DEGs identified in the strain JL-5 under the saline-alkali stress were also enriched into a total of six categories of the metabolic pathways based on the Kyoto Encyclopedia of Genes and Genomes (KEGG) database, including metabolism, genetic information processing, environmental information processing, cell processes, organic systems and human diseases (Figure 4B), with the highest number of genes enriched in metabolism, followed by genetic information processing and environmental information processing. In the category of metabolism, the top three groups of metabolic pathways included carbohydrate metabolism, amino acid metabolism and metabolism of cofactors and vitamins, with the highest number of genes enriched in membrane transport of the genetic information processing category. The results showed that the saline-alkali stress caused significant down-regulation of the cell segregation-related negative regulation gene *SpoVG*, which was consistent with the results of GO annotation, showing that the highest number of genes were annotated into the integral component of the membrane. The saline-alkali stress also altered the expressions of many genes involved in glutamate metabolism, which was consistent with the results of GO annotation, showing that a large number of DEGs were enriched in carbohydrate and amino acid metabolisms. Given the important role that the glutamate metabolism played in the anaerobic respiration to produce lactic acid and the reproduction related metabolic pathways in the strain JL-5, the molecular response of glutamate metabolism to saline-alkali stress was further investigated in the strain JL-5 (below).

#### 2.2.3. Glutamate Metabolism in Response to Saline-alkali Stress in *Priestia aryabhattai* JL-5

The results of the transcriptome analysis revealed the importance of glutamate metabolism in response to saline-alkali stress in the strain JL-5. The KEGG metabolic pathways related to the glutamate metabolism enriched by the DEGs of the strain JL-5 under saline-alkali stress are shown in Table 2, indicating that glutamate metabolism was involved in the response to saline-alkali stress in the strain JL-5 through multiple pathways. Further examinations of these pathways indicated that, in particular, the strain JL-5 was revealed with an enhanced anaerobic respiratory energy supply pathway to produce lactic acid, causing increased expressions of Enolase 1 (ENO1), glyceraldehyde-3-phosphate dehydrogenase (GAPDH), phosphofructokinase of liver type (PFKL), phosphoglycerate kinase 1 (PGK1) and lactate dehydrogenase A (LDHA), induced by hypoxia-inducible factor 1 (HIF-1). Specifically, the expression of ENO1 was directly down-regulated, while the expression of glutamine synthetase (GS) was up-regulated, obtaining more glutamate (Glu) from the tricarboxylic acid (TCA) cycle to convert to glutamine (Gln), which was released extracellularly in the form of GABA through glutamate metabolism. Furthermore, a series of molecular responses through glutamate metabolism were observed in the strain JL-5 under saline-alkali stress. For example, the expressions of both the translation factor LepA and succinate dehydrogenase A (SdhA) were simultaneously affected by the variations in the expression of high temperature protein B (HTPB). In particular, LepA played an important role in the stability and abundance of membrane-related proteins to directly change the membrane structures of the strain JL-5, while SdhA promoted programmed cell death (apoptosis) to maintain cell viability and to promote cell replication in the strain JL-5. Furthermore, the up-regulated expression of glutamate-ammonia ligase (GluL) regulated the phosphate group of the glutamyl phosphate intermediate, which was used by heat shock protein 90 (Hsp90) and receptor-interacting protein kinase 3 (RIPK3), to regulate cell apoptosis, to induce the production of reactive oxygen species (ROS) by the decomposition of glutamine and to ultimately promote cell apoptosis. Moreover, the down-regulation of the Gln ABC transporter substrate-binding protein GlnH caused the decrease in the content of Gln transported to the extracellular area using the ATP transmembrane, ultimately not only decreasing the consumption of ATP but also increasing the content of intracellular Gln, which was more conducive to the decomposition of Gln to produce ROS. In addition, the expression of the permease-like cell division protein (FtsX), which was the same ABC type as the Gln ABC transporter substrate-binding protein GlnH but with no transport function, was up-regulated and used ATP saved by GlnH to promote cell division. These results suggested that strain JL-5 could develop resistance to saline-alkali stress based on glutamate metabolism by reducing its intake of external nutrients, improving the energy supply of anaerobic respiration and ultimately controlling its own apoptosis to rapidly increase the number of viable bacterial cells.

#### 2.2.4. Effect of Saline-alkali Stress on *Priestia aryabhattai* JL-5

**Figure 5:**
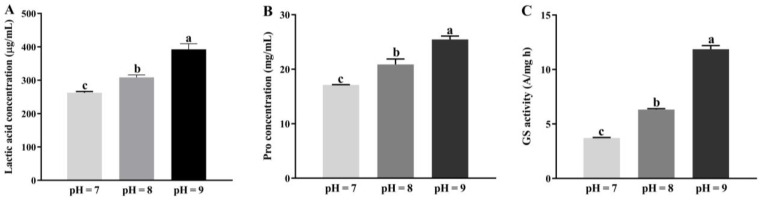
Variations in contents of lactic acid (**A**) and proline (**B**) as well as the glutamine synthetase (GS) activity (**C**) in *Priestia aryabhattai* JL-5 under different saline-alkali stress conditions, i.e., pH = 7 (1.0% NaCl), pH = 8 (1.5% NaCl) and pH = 9 (2.0% NaCl), respectively. Different lowercase letters, a, b and c, indicate the statistical differences based on *p* < 0.05.

The results showed that, in comparison to the strain JL-5 under normal conditions (pH = 7 and 1.0% NaCl), the contents of lactic acid and proline as well as the enzymatic activities of GS were increased by 17.4% and 49.4%, 21.9% and 48.7% and 69.9% and 219% in the strain JL-5 under two saline-alkali stress conditions of pH = 8 with 1.5% NaCl and pH = 9 with 2.0% NaCl, respectively (Figure 5). These results were consistent with those derived from the transcriptome analysis, showing that: (1) the glutamate metabolism was an important pathway involved in the response to saline-alkali stress in the strain JL-5, (2) the strain JL-5 was mainly powered by the anaerobic respiration pathway to produce lactic acid under saline-alkali stress and (3) the increase in proline content and GS activity was caused by the up-regulation of *glnA*, which showed the highest expression in the glutamate metabolism and the most glutamate metabolism-related pathways.

**Figure 6:**
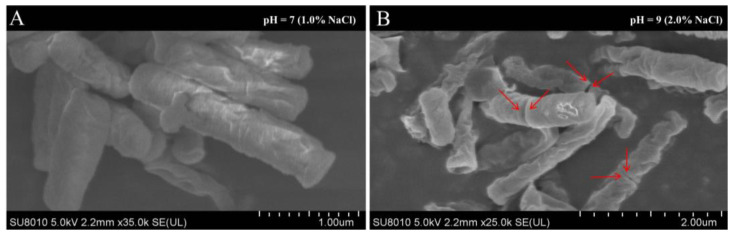
Scanning electron microscopy observations of the *Priestia aryabhattai* strain JL-5 under normal (**A**) and saline-alkali stress (**B**) conditions. The red arrows indicate the locations of asymmetric cell divisions.

The scanning electron microscopy (SEM) observations revealed that under the saline-alkali stress, more cells of strain JL-5 entered the asymmetric cell division (i.e., indicating enhanced bacterial reproduction by fast cell division) and were produced in large quantities (Figure 6). These results were consistent with those derived from the transcriptome analysis, showing that the septation-related negative feedback regulating the gene *SpoVG* involved in the asymmetric cell divisions was down-regulated in the strain JL-5 under saline-alkali stress, causing increased asymmetric cell divisions and the rapid generation of new cells with increased apoptosis and cell replication, i.e., the metabolic component map04217 with *glnA* involved, the metabolic components map04212 and map05134 with *groL* involved and the metabolic component map02010 with *ftsX* involved (Table 2). Again, these results indicated that the strain JL-5 enhanced its own anaerobic respiration energy supply pathway and regulated its own apoptosis and replication to maintain its vitality under saline-alkali stress.

### 2.3. Optimal Ratio of the Strains JL-5 and XW-4 in the Bacterial Solutions and Their Effects on the Growth of Leymus chinensis

**Figure 7:**
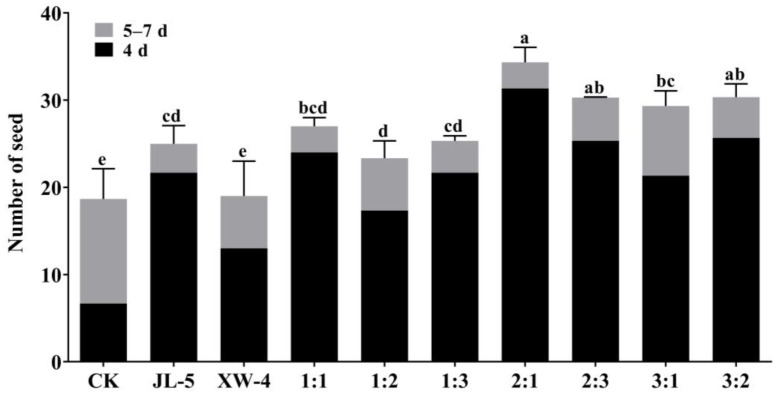
Germination of *Leymus chinensis* seeds treated with combined bacterial solutions of *Priestia aryabhattai* JL-5 and *Staphylococcus pseudoxylosus* XW-4 with different ratios. Control (CK) contains no bacterial solution. Different lowercase letters, a, b, c and d, indicate significant differences based on *p* < 0.05.

The effects of varied ratios of the strains JL-5 and XW-4 in the combined bacterial solution on the germination of *L. chinensis* seeds grown in the saline-alkali soil are shown in Figure 7. The results revealed that most seeds germinated in 4 d, and the highest germination rate of *L. chinensis* seeds was obtained with the ratio of 2:1 between the strains JL-5 and XW-4 in the bacterial solution applied into the saline-alkali soil samples. Therefore, the optimal ratio of 2:1 was used in the following experiments. From day 5 to day 7, the highest germination rate was achieved in the control group, followed by the combined treatment of the strains JL-5 and XW-4 with a ratio of 3:1. In 8 d and thereafter, seed germination was rarely observed.

**Figure 8:**
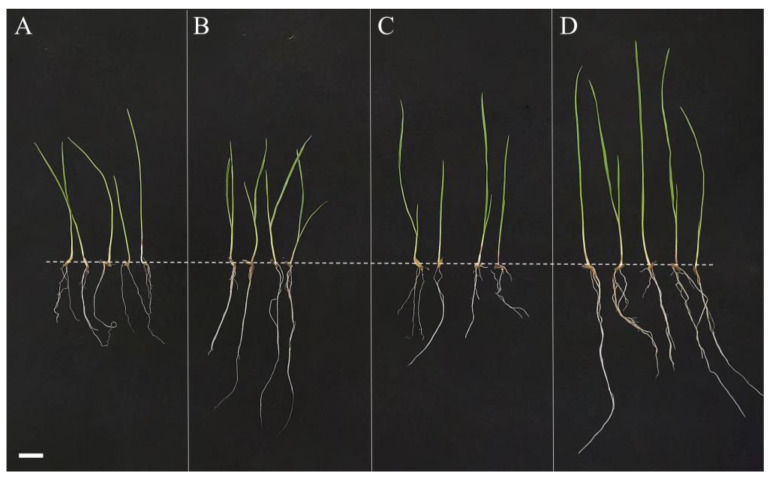
Phenotypic variations in *Leymus chinensis* plants treated with (**A**) no bacterial solution, (**B**) bacterial solution of *Priestia aryabhattai* JL-5, (**C**) bacterial solution of *Staphylococcus pseudoxylosus* XW-4 and (**D**) combined bacterial solutions of both JL-5 and XW-4. Scale bar = 1 cm. The dashed line indicates the location of the seeds, showing the roots and leaves below and above the line, respectively.

The seedlings were allowed to grow for 15 d until tillering to determine the contents of SOD, POD, CAT, MDA and proline (Figure 8). The results showed that the plants of *L. chinensis* treated with combined bacterial solutions entered the tillering stage in a shorter time than the plants without the treatment of bacterial solutions (Table 3). It was noted that the roots and time required to enter the tillering stage of *L. chinensis* treated with the strain JL-5 were significantly longer and shorter, respectively, than those of plants without the treatment of bacterial agents or those treated with XW-4. The roots and leaves of *L. chinensis* treated with a mixed bacterial solution of both JL-5 and XW-4 were significantly longer than those of plants either treated with one bacterial strain alone or untreated with bacterial solutions (control), while the time required to enter tillering was shorter than that of those treated with strain JL-5 (Table 3).

**Figure 9:**
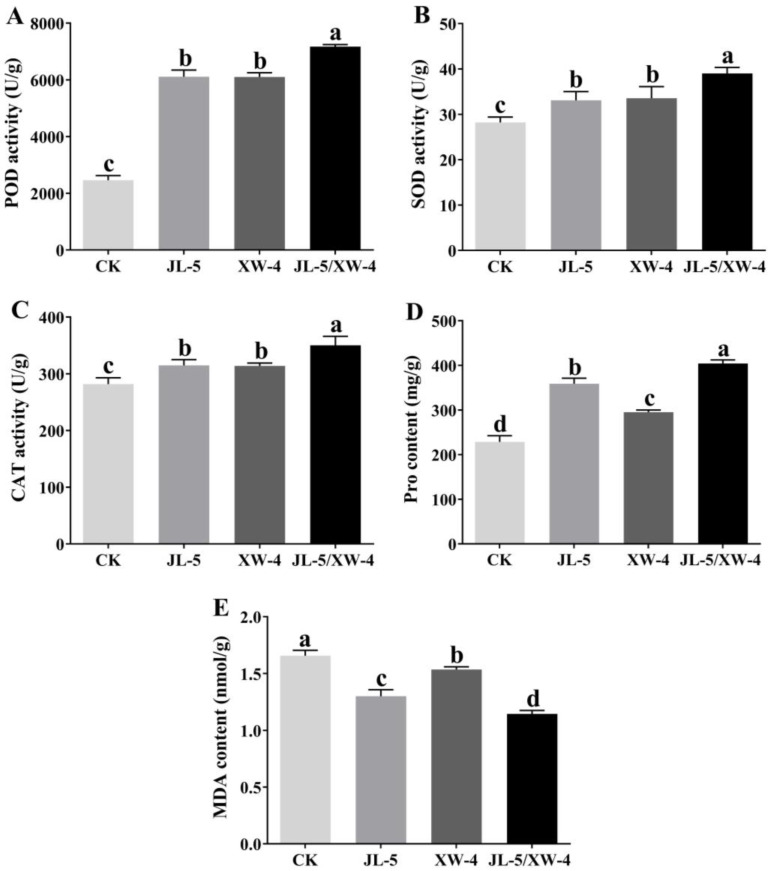
Variations in the contents of (**A**) peroxidase (POD), (**B**) superoxide dismutase (SOD), (**C**) catalase (CAT), (**D**) proline (Pro) and (**E**) malondialdehyde (MAD) in plants of *Leymus chinensis* treated with separate and combined bacterial solutions of *Priestia aryabhattai* JL-5 and *Staphylococcus pseudoxylosus* XW-4. Different lowercase letters, a, b, c and d, represent the significant differences based on *p* < 0.05.

The contents of the enzymes (i.e., POD, CAT and SOD) of the oxidase system as well as the MDA and proline in plants of *L. chinensis* under the saline-alkali stress treated with strains JL-5 and XW-4 are shown in Figure 9. The results revealed the highest activities of the antioxidant enzymes and the contents of proline in *L. chinensis* treated with the combined bacterial solution, followed by those treated with one bacterial strain alone and untreated groups. It was worth noting that the content of proline in *L. chinensis* grown in soil treated with the strain JL-5 was higher than that in those treated with strain XW-4. The content of MDA in plants of *L. chinensis* treated with a mixed bacteria solution of both JL-5 and XW-4 was significantly decreased in comparison with that of those treated with one bacterial strain alone. The highest content of MDA was detected in the control group without the treatment of the bacterial solution.

### 2.4. Effects of Bacterial Solutions on the Soil Nutrients and Microbial Community

#### 2.4.1. Effects of Bacterial Solutions on Available Nitrogen, Phosphorus and Potassium and Organic Matter in Soil

**Figure 10:**
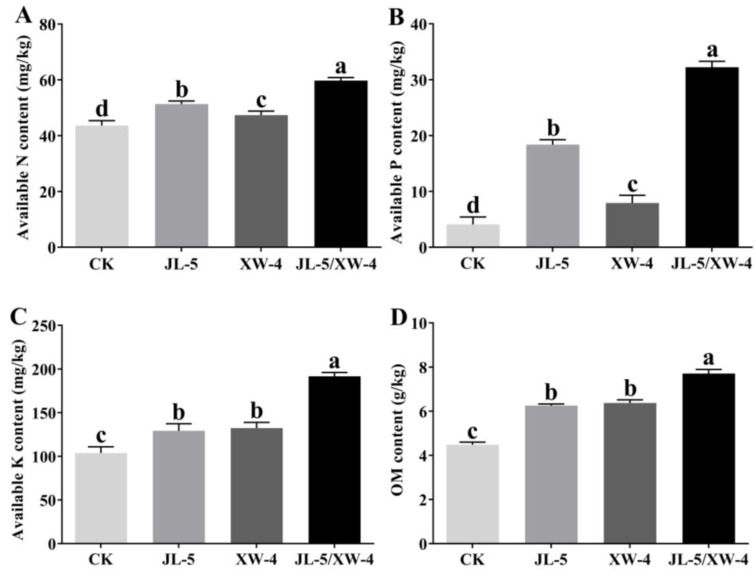
Variations in the soil nutrients of (**A**) nitrogen (N), (**B**) phosphorus (P) and (**C**) potassium (K) and (**D**) organic matter (OM) in soils treated with individual and combined bacterial solutions of *Priestia aryabhattai* JL-5 and *Staphylococcus pseudoxylosus* XW-4. Control (CK) contains no bacterial solutions. Different lowercase letters, a, b, c and d, represent the significant differences based on *p* < 0.05.

The highest contents of various nutrients, i.e., available nitrogen (N), phosphorus (P) and potassium (K), and organic matter were revealed in the saline-alkali soil treated with the combined bacterial solution, followed by those treated with one bacterial strain alone and the untreated groups (Figure 10). It was noted that the contents of P were significantly increased in the soils treated with either the strain JL-5 or the combined bacterial solution of both JL-5 and XW-4. However, the content of P was increased less in the treatment of the strain XW-4 than in the treatment of the strain JL-5, suggesting a synergistic effect between the strains JL-5 and XW-4 on the increased growth of the strain JL-5 in the saline-alkali soils.

#### 2.4.2. Effects of the Combined Bacterial Solution on Soil Microbial Diversity

QIIME2 (2019.4) was used to calculate the total number of bacterial operational taxonomic units (OTUs) in the samples at the given sequencing depths of 16S rRNA, with the rarefaction curves of six control and six experimental groups of soil samples shown in Figure S1. The 16S rRNA sequence similarity threshold of 97% was used to establish the bacterial OTUs. These results showed that, as the sequencing depth was increased, the number of OTUs in both the control and experimental groups tended to be saturated. At the same sequencing depth, the number of OTUs of the experimental groups was larger than that of the control groups, indicating that the sample size for sequencing was appropriate, with the sequencing data generated being suitable for the subsequent analyses.

**Figure 11:**
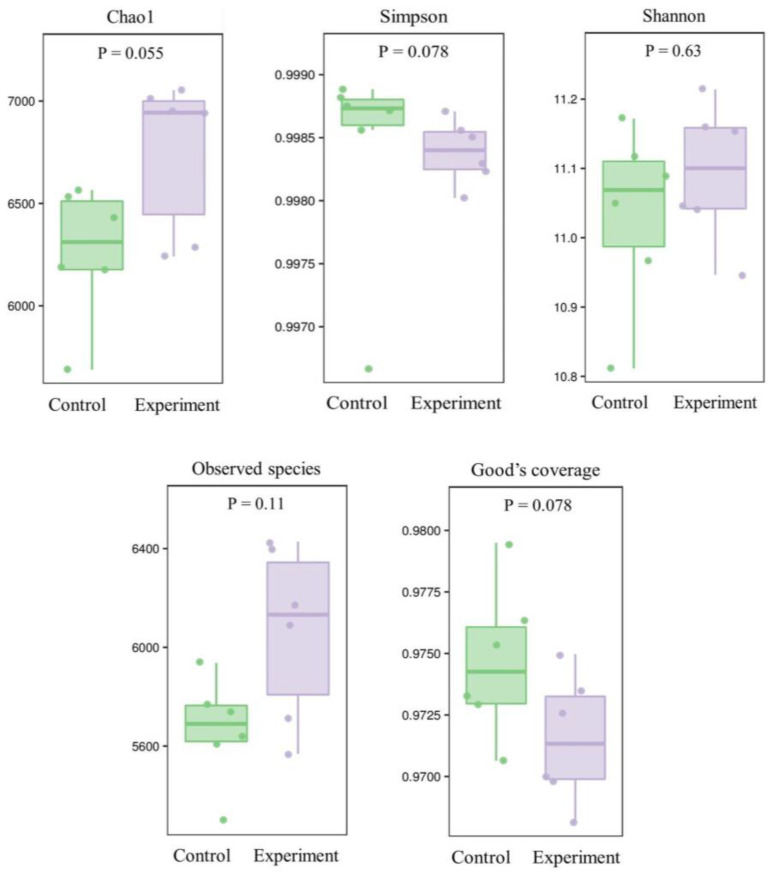
Microbial diversity based on the alpha diversity indices (i.e., Chao1, Simpson, Shannon, observed species and Good’s coverage) in soils of six control groups treated with no bacterial solutions and six experimental groups treated with the bacterial mixture of *Priestia aryabhattai* JL-5 and *Staphylococcus pseudoxylosus* XW-4.

The microbial community structures in the soils treated with the combined bacterial solution (i.e., the experimental groups) and in the untreated soils (i.e., the control groups) were evaluated using the 16S rRNA amplicon sequencing. After the removals of duplicated and chimeric sequences, the high-quality 16S rRNA sequences obtained from each sample were used for subsequent analyses (Figure 11; Table S2). The results of alpha diversity analysis revealed no significant differences in species richness between the control and experimental groups.

Based on the Bray–Curtis distance and UniFrac distance, the beta diversity analysis, i.e., the principal coordinate analysis (PCoA), was performed using QIIME2 (2019.4) to reveal the variations among the soil samples of the control and experimental groups. The results showed that the soil samples of the control groups and experimental groups were evidently clustered into two distinct groups, respectively (Figure S2).

**Figure 12:**
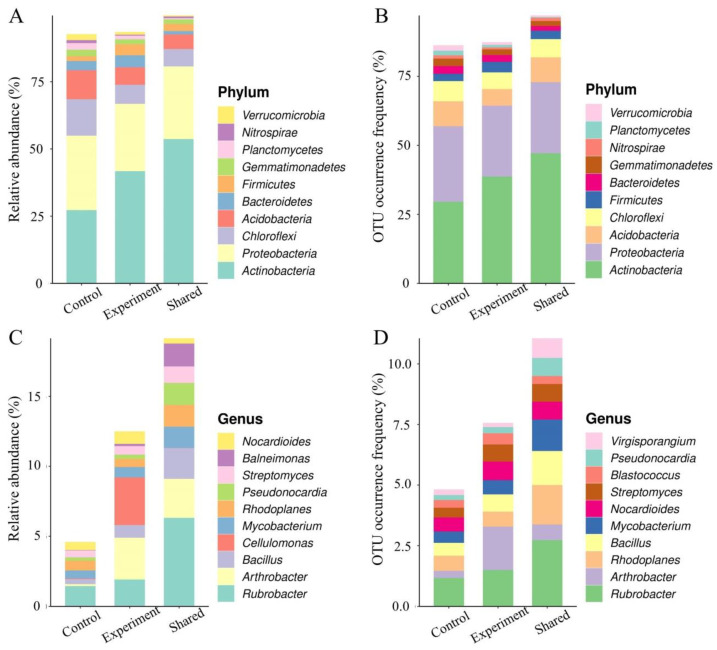
Effects of the combined bacterial solution of both *Priestia aryabhattai* JL-5 and *Staphylococcus pseudoxylosus* XW-4 on soil microbial abundance and diversity. (**A**,**B**) Relative abundance and relative OTU frequency of the top 10 bacterial phyla in the experimental and control groups and those shared between both groups. (**C**,**D**) Relative abundance and relative OTU frequency of the top 10 bacterial genera in the experimental and control groups and those shared between both groups.

A total of 13,748 (38.09%) and 15,263 (42.29%) OTUs were uniquely identified in the experimental and control groups, respectively, sharing 7083 (19.62%) OTUs in both groups. The results showed that, at the phylum level, the relative abundances of Actinobacteria, Bacteroides and Firmicutes and the OTU occurrence frequencies of Actinobacteria and Firmicutes were significantly increased in the experimental groups (Figure 12A,B). At the genus level, the relative abundances of *Rubrobacter, Arthroactor, Bacillus, Cellulomonas* and *Nocardioides* and the OTU occurrence frequencies of *Rubrobacter, Arthroactor, Bacillus, Mycobaterium, Nocardioides, Streptomycetes* and *Blastococus* in the experimental groups were significantly higher than those of the control groups (Figure 12C,D).

**Figure 13:**
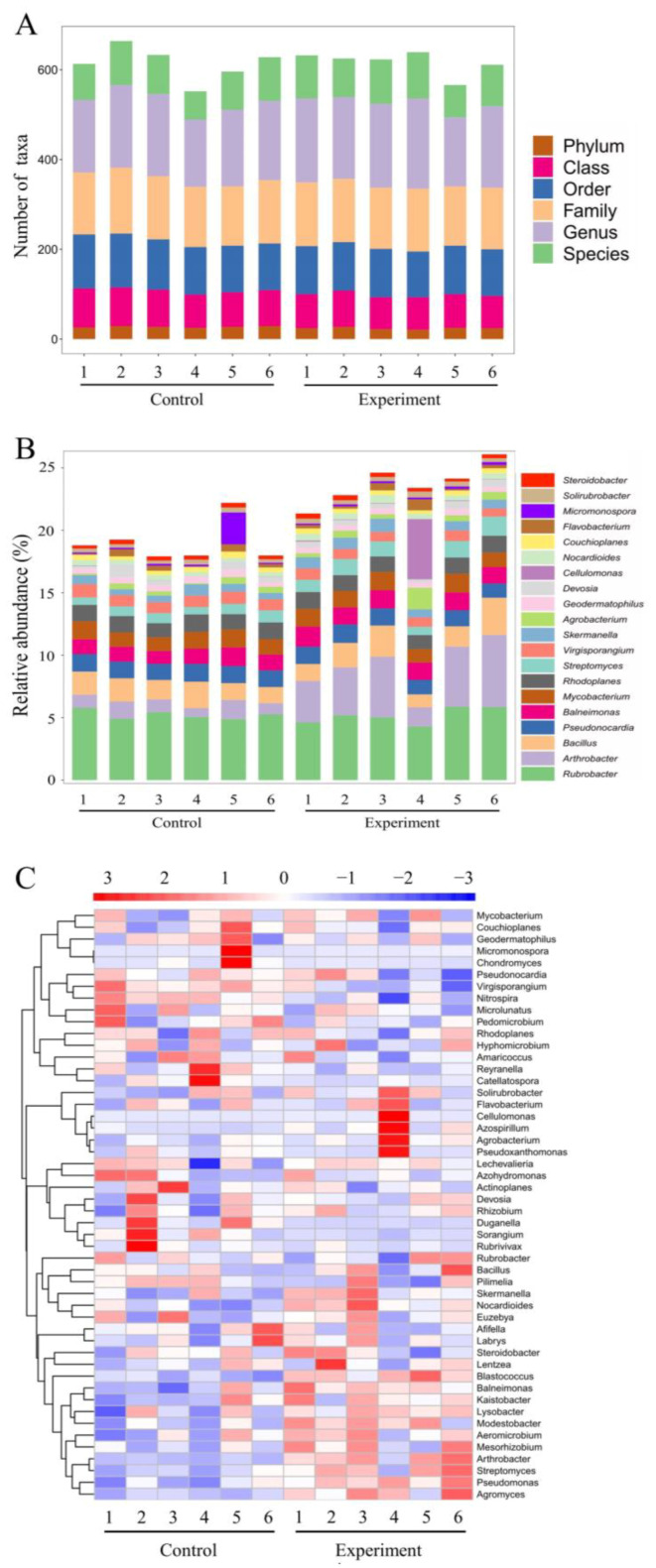
Effects of combined bacterial solutions of both *Priestia aryabhattai* JL-5 and *Staphylococcus pseudoxylosus* XW-4 on soil microbial structures, showing the number of taxa at various taxonomic levels (**A**), the relative OTU abundance at the genus level (**B**) and the clustering heatmap of OTUs at the genus level (**C**).

Significant differences were observed in the total number of taxa at various taxonomic levels between the experimental and control groups (Figure 13A). In particular, the top 20 genera showing the highest variations in the relative OTU abundance are shown in Figure 13B, including *Trodider, Sotirubrobacter*, *Micromonospora*, *Flavobacterium*, *Couchioplanes*, *Nocardioides*, *Cellulomonas*, *Devosia*, *Geodermatophilus*, *Agrobacterium*, *Skermanella*, *Virgisporangium*, *Streptomyces*, *Rhodoplanes*, *Mycobacterium*, *Barneimonas*, *Pseudonocardia*, *Bacillus*, *Arthrobacter* and *Rubrobacter*. The heatmap based on the association analysis of the top 50 genera revealed the significant increase in their relative abundance in a total of 28 genera (Figure 13C), including *Agromyces, Pseudomonas*, *Streptomyces*, *Arthrobacter*, *Mesorhizobium*, *Aeromicrobium*, *Modestobacter*, *Lysobacter*, *Kaitobacter*, *Blastococcus*, *Lentzea*, *Steroidobacter*, *Labrys*, *Euzebya*, *Nocardioides*, *Skermanella*, *Bacillus*, *Rhizobium*, *Hyphomicrobium*, *Lechevalieria*, *Azospirllum*, *Pseudoxanthomonas*, *Solirubrobacter*, *Flavobacterium*, *Cellulomonas*, *Agrobacterium*, *Mycobacterium* and *Balneimonas*. The significant decrease in the relative abundance was observed in the other 22 genera, including *Chondromyces*, *Actinoplanes*, *Azohydromonas*, *Rubrivivax*, *Sorangium*, *Pilimelia*, *Catellatospora*, *Pedomicrobium*, *Reyranella*, *Amaricoccus*, *Microlunatus*, *Duganella*, *Afifella*, *Nitrospira*, *Micromonospora*, *Couchioplanes*, *Devosia*, *Geodermatophilus*, *Virgisporangium*, *Rhodoplanes*, *Rubrobacter* and *Pseudonocardia* (Figure 13C).

The top 15 marker taxa identified in the taxonomic variations between the experimental and control groups included 2 taxa of *unidentified Rubrobacteraceae*, *Arthrobacters*, *unclassified Micrococcaceae*, *Pseudomonas* and *unidentified Rhodospirillales* and 1 taxon of *Streptomyces*, *Flavobacterium*, *unidentified Acidimicrobiales*, *unidentified Hypomicrobiaceae*, *Balneimonas* and *unidentified ellin6529* (Figure S3A). In comparison to the control groups, the experimental groups showed strong associations or interactions among a total of 13 taxa, including *Arthrobacter*, *Bradyrhizobiaceae*, *Solidrubrobacterales*, *Arthrobacter*, *Geinm*, *Proteobacteria*, *Bacillus*, *Gaiellaceae*, *Geodermatophilaceae*, *Skermanella*, *Rhizobiales*, *Balneimonas* and *Peaodonocardiaceae* (Figure S3B). These results showed that the combined bacteria solutions could significantly improve the richness of soil microorganisms, while both the strain JL-5 and *Bacillus* were closely related to each other, showing extremely similar chemical and physiological properties, and they were closely associated with *Arthrobacter*, *Bradyrhizobiaceae*, *Solidubrobacteres*, *Geinm*, *Proteobacteria*, *Solidubrobacteres*, *Gaiellaceae*, *Geodermatophilaceae*, *Skermanella*, *Rhizobiales*, *Balneimonas* and *Peaodonocardiaceae* in soil. These taxa were also closely related to the marker taxa in the taxonomic variations between the experimental and control groups as well as the bacterial taxa that were significantly up-regulated in the experimental groups, i.e., close associations were revealed among the relatively dominant taxa showing the significant intersections with the taxa of high importance in the soils. These results evidently revealed the significant effects of combined bacterial solutions on the change in soil microbial taxonomic structures.

#### 2.4.3. Effects of the Combined Bacterial Solution on Soil Microbial Metabolic Pathways

**Figure 14:**
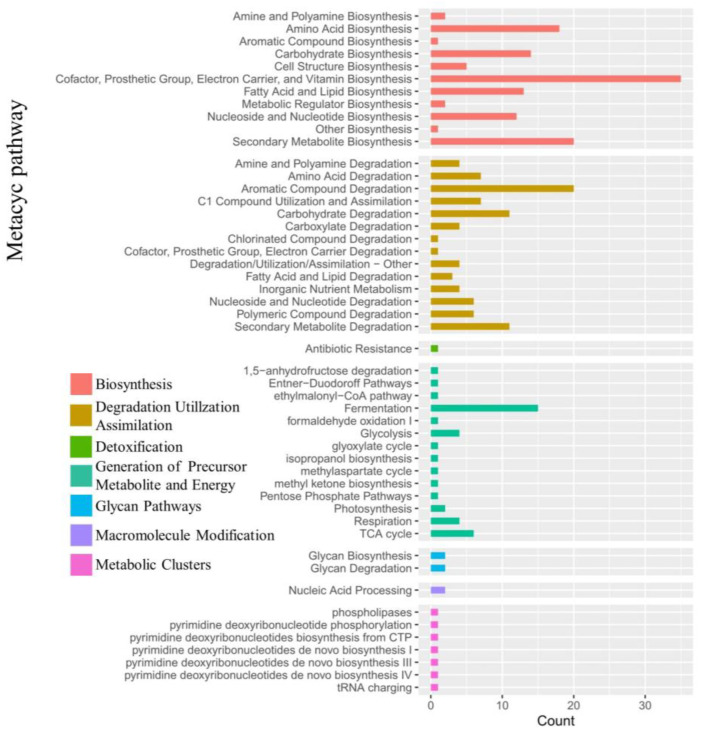
Enrichment of the microbial metabolic pathways based on the MetaCyc Metabolic Pathway Database in the soils treated with bacterial solutions of both *Priestia aryabhattai* JL-5 and *Staphylococcus pseudoxylosus* XW-4.

The enrichment analysis of the 16S rRNA data was performed using PICRUSt2 based on the MetaCyc Metabolic Pathway Database, with a group of seven categories of metabolic pathways significantly enriched, including biosynthesis, degradation utilization assimilation, detoxification, generation of the precursor metabolite and energy, glycan pathways, macromolecule modification and metabolic clusters (Figure 14). The highest numbers of genes were enriched in the categories of biosynthesis and degradation utilization assimilation, followed by detoxification. In particular, many metabolic pathways in the experimental groups were significantly enhanced by their interactions with the strains JL-5 and XW-4, e.g., the pathways involved in amino acid synthesis, cofactor electron carrier synthesis, fatty acid and lipid synthesis, nucleotide synthesis, fermentation and the TCA cycle.

**Figure 15:**
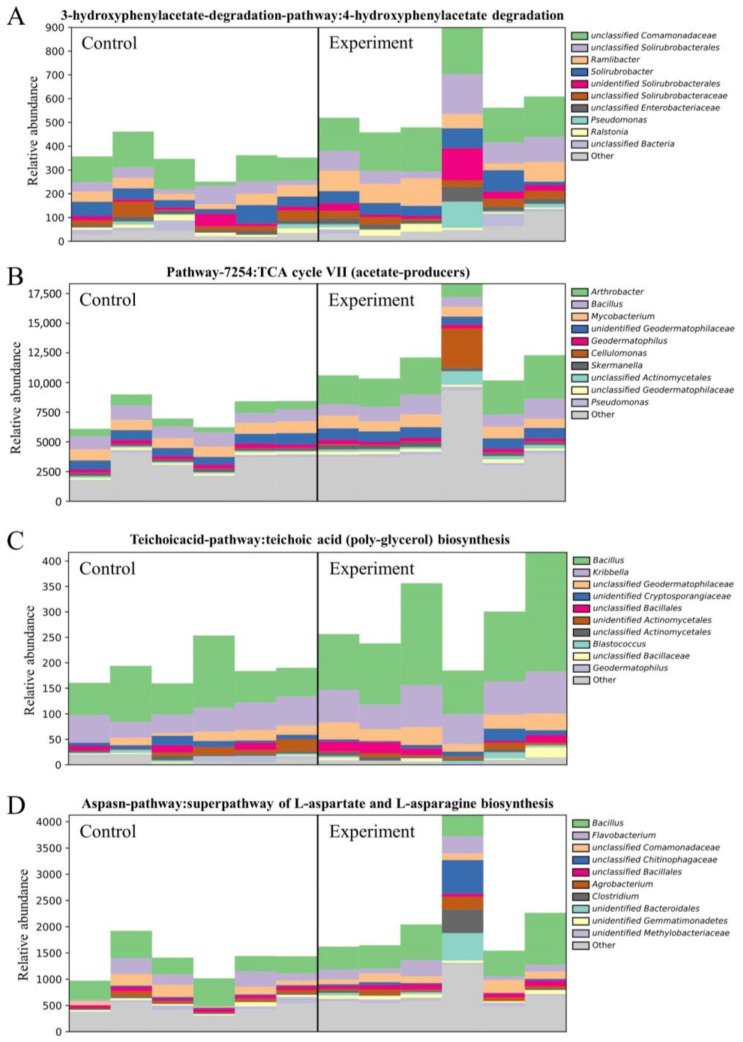
Variations in four significantly enriched metabolic pathways based on the MetaCyc Metabolic Pathway database of the top 10 bacterial genera in soils of six control groups treated with no bacterial solutions and six experimental groups treated with bacterial solutions of both *Priestia aryabhattai* JL-5 and *Staphylococcus pseudoxylosus* XW-4.

The significant enhancements were detected in four metabolic pathways in soil samples treated with bacterial solutions of both JL-5 and XW-4 (Figure 15), including the (1) 3-hydroxyphenylacetate degradation pathway: 4-hydroxyphenylacetate degradation, (2) pathway-7254: TCA cycle VII (acetate-producers), (3) teichoicacid-pathway: teichoic acid (poly-glycerol) biosynthesis and (4) aspasn-pathway: super-path of L-aspartate and L-asparagine biosynthesis (Figure 14). Among the taxa with a high relative abundance in the experimental groups, e.g., *Bacillus*, *Kribbella* and *Arthrobacter*, *Bacillus* was observed with the greatest variations in the synthesis of teichoic acid and the metabolism of aspartic acid under the treatment of combined bacterial solutions.

## 3. Discussion

### 3.1. Alleviated Saline-alkali Stress by Priestia aryabhattai JL-5 via Glutamate Metabolism

To date, the biological controls using microorganisms to improve the saline-alkali soils are generally low-cost, fast and efficient bioremediation strategies. Studies have shown that by isolating the saline-alkali-tolerant microorganisms from the saline-alkali soils, the understanding of the molecular mechanisms regulating the saline-alkali tolerance and the interactions between microbiota and local plants in soils could help improve the saline-alkali soil environments [30]. As single-celled organisms, bacteria have to obtain the nutrients externally to ensure their own survival, growth and reproduction [31]. Therefore, the growth and taxonomic components of bacteria are directly impacted under the external stresses [32], suggesting that the efficient allocation of the energy required by bacteria to adapt to the adverse environments and to quickly ensure their survival as well as the local microbiota directly determine the adaptive strategies of bacteria to the stressed environments [33,34].

**Figure 16:**
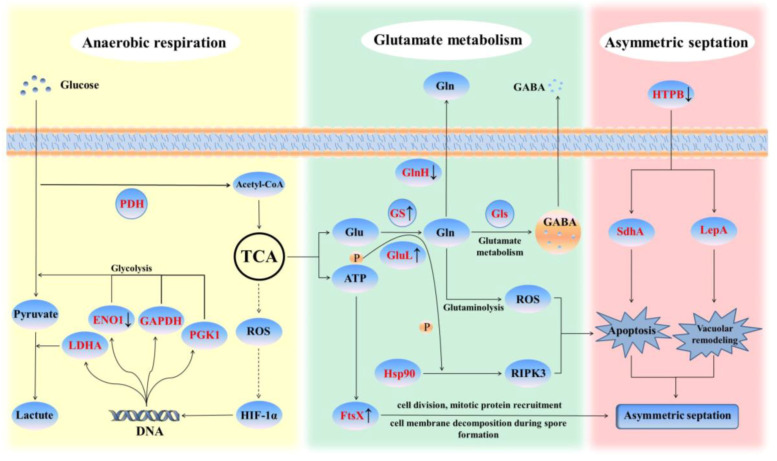
Schematic diagram of the molecular response to saline-alkali stress in *Priestia aryabhattai* JL-5. The enzymes highlighted in red are revealed with altered expression levels in this study, with symbols “↑” and “↓” indicating up-regulation and down-regulation, respectively. FtsX, permease-like cell division protein; PDH, pyruvate dehydrogenase; ENO1, enolase 1; GABA, γ-aminobutyric acid; GAPDH, glyceraldehyde-3-phosphate dehydrogenase; Gln, glutamine; Gls, glutaminase; Glu, glutamate; GlnH, glutamine ABC transporter substrate-binding protein; GluL, glutamate-ammonia ligase; GS, glutamine synthetase; HIF-1α, hypoxia-inducible factor-1α; Hsp90, heat shock protein 90; HTPB, high temperature protein B; LDHA, lactate dehydrogenase A; LepA, translation factor LepA; P, phosphate group; PGK1, phosphoglycerate kinase 1; RIPK3, receptor-interacting protein kinase 3; ROS, reactive oxygen species; SdhA, succinate dehydrogenase A.

Studies have shown that bacteria usually respond to saline-alkali stress by changing their membrane permeability to stimulate the proton pump switch and to improve their metabolic activities [35,36,37,38]. In our study, the results of transcriptome sequencing showed that the strain JL-5 would adopt a unique strategy in response to saline-alkali stress, with several metabolic pathways involved, including anaerobic respiration, glutamate metabolism and asymmetric septation (Figure 16). In particular, with the excessive external supply of glucose, the strain JL-5 was stimulated to produce pyruvate dehydrogenase (PDH), which was a metabolic enzyme involved in the conversion of pyruvate to acetyl coenzyme A. Studies have shown that PDH is generally used by cells to regulate energy metabolism to achieve cellular homeostasis [39], while the changes in PDH could alter the interactions among glycolysis, the TCA cycle and anaerobic respiration [40], ultimately causing the hypoxia inducible factor HIF-1 to change the expressions of ENO1, GAPDH, PFKL, PGK1 and LDHA, with LDHA showing a high affinity for pyruvic acid and participating in the conversion of pyruvic acid to lactic acid [41,42]. Due to the enhanced energy supply pathway for providing lactic acid to the bacteria, the function of the strain JL-5 to obtain external nutrients would be limited, while the reduced content of ROS in cells would affect the expressions of SOD-3 and CTL-1/2, ultimately improving the resistance of the strain JL-5 to stress to maintain its cellular activities [43,44]. Furthermore, studies have shown that the expressions of both ENO1 and Hsp60 are directly inhibited under the saline-alkali conditions, while the inhibition of ENO1 could reduce the energy supply obtained by cells through glycolysis, causing increased pyruvate lactate energy supply and further activating the chain reaction triggered by the reduction in ROS in cells [45]. As two types of chaperonins, both GroEL (*E. coli* Hsp60 or heat shock protein family D member 1 (HspD1)) and Hsp60 share highly similar molecular structures and functions, e.g., the inhibition of Hsp60 could cause cell apoptosis and improve resistance [46]. These studies suggested that the bacterial cells with strong physiological activities and metabolic functions could survive the apoptosis to enhance the tolerance of the bacterial population to saline-alkali stress. Because PDH could affect the TCA cycle by altering the production of acetyl coenzyme A, the TCA, in turn, enables glutamate-ammonia ligase (GS), which is directly increased in its expression by saline-alkali stress, to obtain more glutamate (Glu) to generate glutamine (Gln), which is converted to γ-aminobutyric acid (GABA) under the joint influence of both glutaminase (GLS) and glutamate metabolisms [47]. Under this strategy, the strain JL-5 not only decreased its energy metabolism to reduce the external energy intake but also maintained its vitality through the feedback of this energy metabolism in the saline-alkali environments. In our study, the results of transcriptome sequencing showed that the strain JL-5 under the saline-alkali stress could enhance the energy supply pathway of anaerobic respiration, with the gene *glnA* showing the highest up-regulated expression, further regulating the glutamate metabolism via GS, which was closely related to proline synthesis (Table 2). These results were consistent with those previously reported [48,49,50,51], showing that *glnA* was closely related to the production of proline and GS involved in the glutamate metabolism. It is commonly known that proline is one of the main substances generated by bacteria involved in the stress alleviation in plants [52]. Our results showed that as the levels of saline-alkali stress were increased, the contents of proline and lactic acid as well as the enzymatic activities of GS in the strain JL-5 were significantly increased, with most of the genes showing altered expressions involved in carbohydrate metabolism and amino acid metabolism, suggesting the enhanced glutamate metabolism in the strain JL-5 under the saline-alkali stress (Figure 16). These results were consistent with the expression patterns of metabolic pathways in the strain JL-5 (Table 2)—in particular, the high expression level of *glnA*. These results strongly indicated that the glutamate metabolism played important roles in connecting multiple metabolic pathways in response to saline-alkali stress in the strain JL-5.

### 3.2. Physiological Variations in Priestia aryabhattai JL-5 in Response to Saline-alkali Stress

Our results showed that a series of molecular responses were initiated in the strain JL-5 to maintain its cell viability and to promote bacterial cell replication under the saline-alkali conditions. Studies have shown that as a type of multifunctional chaperone protein, the expression of HTPB is directly affected by the saline-alkali stress, ultimately entering the cell as an effector of the cell signaling pathway [53]. These results are consistent with the findings revealed in our study, showing that the expression of HTPB was decreased in the strain JL-5 under the saline-alkali stress. Furthermore, studies have shown that LepA plays an important role in the stability and richness of membrane-related proteins [54], while SdhA not only participates in the TCA cycle but also promotes apoptosis by affecting the caspase metabolism [55]. In our study, the results showed that LepA was affected by the down-regulated expression of HTPB, suggesting that both LepA and SdhA were involved in bacterial cell apoptosis, probably through the caspase metabolism. These studies suggested that the saline-alkali stress caused the membrane changes and, ultimately, the cell apoptosis in the strain JL-5 to maintain its cell vitality and promote cell replication. Moreover, studies have shown that both Hsp90 and RIPK3 are involved in the regulation of cell apoptosis through phosphorylation [56], while the expression of Gln via GluL would release the phosphate group of the glutamyl phosphate intermediate [57]. In this process, the down-regulated expression of GlnH is conducive to the accumulation of Gln in the cells, while the up-regulated expression of GluL could provide phosphate groups for Hsp90 and RIPK3, which are conducive to the decomposition of Gln, the induction of ROS and the promotion of cell apoptosis [58]. These studies are consistent with the findings revealed in our study, showing that the expression of GluL in the strain JL-5 was up-regulated under saline-alkali stress, and the expression of Hsp90 was also affected, while GluL provided phosphate groups for Hsp90 to produce a large amount of Gln, and the down-regulation of the expression of GlnH would also reduce the Gln transported to the outside of the cell and increase the content of Gln inside the cell, ultimately inducing the production of ROS and promoting bacterial cell apoptosis. Our results showed that under the saline-alkali stress, the membrane activities were enhanced in the strain JL-5, indirectly regulating its own apoptosis to ensure the vitality of the bacterial population through the TCA cycle and glutamate biosynthesis pathways, ultimately stimulating its own replication.

Furthermore, our results showed that, due to the significant down-regulation of the negative feedback gene *SpoVG*, which is involved in the regulation of asymmetric septation, a large number of bacterial cells of the strain JL-5 would be maintained at the segregation stage to replicate, as previously reported [59]. These results suggested that the large number of cells in both replication and apoptosis in the strain JL-5 were maintained by the regulation of asymmetric cell division. The asymmetric cell division was first discovered by Oh et al. in 1973, revealing that the bacterial cells of *Bacillus* formed an asymmetric diaphragm, i.e., the newly formed cell wall, which was thicker than the normal cell wall [60]. At the end of the asymmetric septation, one end forms a mother cell, and the other end forms a spore. Studies have shown that as the expression of *SpoVG* is inhibited, the bacteria would enter and complete the asymmetric septation faster than normal cell divisions [59]. Moreover, studies have shown that *SpoVG* plays important roles in regulating spore formation and biofilm formation in *Bacillus cereus* based on the genetic knockout of *SpoVG*, providing a strategy for bacteria to adapt to the adverse environments [61]. These studies were consistent with the results derived from the transcriptome analysis in our study, showing that the expression of *SpoVG* in the strain JL-5 was down-regulated under saline-alkali stress, affecting the synthesis of biofilm in bacterial cells. Furthermore, studies have shown that the up-regulated expression of the permease-like cell division protein FtsX is involved in the spatial and temporal regulations of the mitotic protein recruitment, cell division and cell membrane decomposition during spore formation [62], while the positive expressions of these characteristics are consistent with the physiological characteristics of cells during asymmetric septation. These results were consistent with the findings revealed in our study, i.e., FtsX expression was significantly up-regulated in the strain JL-5 under the saline-alkali stress.

Moreover, our SEM observations showed that more cells of the strain JL-5 entered the asymmetric septation under saline-alkali stress than the bacterial cells under normal conditions, suggesting that the strain JL-5 developed this adaptive strategy in response to the saline-alkali stress. Therefore, the strain JL-5 entered into asymmetric septation to enhance the cell viability and increase the number of cells under saline-alkali stress, which was conducive to the generation of more metabolites, e.g., lactic acid, proline and GABA. The production of more lactic acid was beneficial to neutralizing the high pH level of saline-alkali soil, while the enhanced production of proline and GABA was beneficial to alleviating the saline-alkali stress of plants in the soil.

### 3.3. Improved Saline-alkali Soils by Combined Bacterial Solutions Promoting the Interaction of Leymus chinensis and Other Microorganisms

Our results showed that under the saline-alkali stress, the strain JL-5 could alter the expression levels of some metabolites, such as proline and GABA, and interact with other taxa in the soil microbiota. These results were consistent with those reported previously, showing that GABA could induce the rhizobia bacteria [63,64]. Similarly, our results showed that one of the marker taxa identified in the experimental group (i.e., unidentified Hypomicrobiaceae) belonged to the photosynthetic bacterial order Rhizobiales, while the large number of bacteria of *Arthrobacter* produced a large amount of acetate. Studies have shown that as a type of substance closely involved in plant environmental stress responses, GABA is involved in the saline tolerance in tomato plants as the accumulation of ROS is increased [65]. In our study, the increased contents of proline in the indicator plants of *L. chinensis* and the increased contents of available P in the soil were directly caused by the application of the bacterial solutions containing both JL-5 and XW-4, whereas the treatment of the strain JL-5 alone achieved significantly decreased contents of these substances. Furthermore, the content of available P in the saline-alkali soil treated with the strain JL-5 alone was significantly higher than those in the control group and in the saline-alkali soil treated with the strain XW-4 alone. However, the content of available P in saline-alkali soil treated with a mixture of both JL-5 and XW-4 was higher than that of the treatment of the strain JL-5 alone. These results evidently indicated the importance of the interactions among the microorganisms, with the changes in the content of available P mainly caused by the treatment of the strain JL-5, while the interaction between the strains JL-5 and XW-4 further promoted the increase in the content of available P in soils. These results were in accordance with the results previously reported, confirming the importance of the interactions among soil microorganisms [66].

Our results revealed the significance of bacterial solutions in altering the microbial community richness in the saline-alkali soil, showing the interactions among a variety of bacteria with varied relative abundances in the experimental groups and the bacteria of the same genera in the treatment of the combined bacterial solution. It is well known that the variations in the metabolic pathways in soils could be predicted by the functional abundance based on the 16S rRNA sequence abundance in the samples [67]. These applications are currently common experimental strategies in various investigations of the microbial community structure and metabolic functions. For example, studies have shown that the comparative analysis of 16S rRNA and the transcriptome are performed to explore the variations in the metabolic pathways to explore the effects of feed on microbial metabolic pathways in bovine rumen [67]. These results are consistent with the findings revealed in our study, showing the enhanced metabolic pathways involved in amino acid synthesis, cofactor electronic carrier synthesis, fatty acid and lipid synthesis, nucleotide synthesis, fermentation and the TCA cycle. In particular, the comparative analysis of the relatively dominant bacteria showing four significantly enhanced metabolic pathways, i.e., 3-hydroxyphenylacetate degradation pathway: 4-hydroxyphenylacetate degradation, pathway-7254: TCA cycle VII (acetate-producers), teichoicacid-pathway: teichoic acid (poly-glycerol) biosynthesis and aspasn-pathway: super-path of L-aspartate and L-asparagine biosynthesis, revealed that these main bacteria (i.e., *Ramlibacter*, *Arthrobacter*, *Bacillus* and *Kribbella*) were largely the same as those showing significant variations between the experimental and control groups. It is worth noting that the strain JL-5 mainly interacted with *Arthrobacter* in soils to produce acetate via the TCA cycle to ultimately alleviate the saline-alkali stress and to improve the soil environments. Similarly, previous studies showed that acetate produced by bacteria would improve the saline-alkali soil property [68], while the aspartic acid could produce glutamic acid through transamination [69,70], and the teichoic acid was an important structural component of the cell wall of Gram-positive bacteria [71].

Furthermore, the bacteria that mainly produced teichoic acid in the experimental group and participated in the aspartic acid metabolic pathway belonged to *Bacillus*, which shared largely the same biochemical properties as those of the strain JL-5. These results suggested that the enhanced teichoic acid metabolism was probably caused by the response of Gram-positive bacteria to osmotic stresses, e.g., salt stress, as previously reported. For example, studies have shown that *Staphylococcus aureus* is rich in teichoic acid, causing the formation of thickened cell walls, which are less prone to rupture under the attack of a mixture of external contaminants [72]. However, since *Bacillus* represented the main effective bacteria responsible for the improved metabolism of teichoic acid, its function was probably to perform massive proliferation [73]. Moreover, the 4-hydroxyphenylacetate is a product derived from lignin decomposition [74] and could be used as a carbon source for microbial growth [75]. These results strongly suggested that the treatments of bacterial solutions of both JL-5 and XW-4 significantly increased not only the contents of organic matters in soil by degrading corn straw (by strain XW-4) but also the interactions with other bacterial taxa of the local soil microbiota. In this process, the strain JL-5 obtained more Glu through the aspartic acid pathway by the up-regulated expressions of *gatA*, *gatB* and *gatC* involved in the metabolic pathway map00970 to maintain the glutamate metabolism and its massive cell proliferation in response to saline-alkali stress (Table 2; Figure 16).

**Figure 17:**
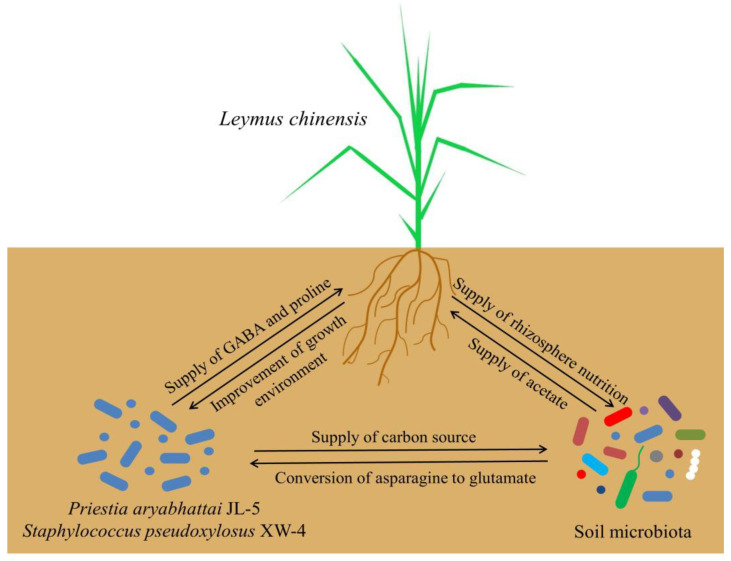
Schematic diagram of the synergetic interactions among *Priestia aryabhattai* JL-5, *Staphylococcus pseudoglossus* XW-4, plants of *Leymus chinensis* and soil microbiota. GABA, γ-aminobutyric acid.

The synergistic effects of the strains JL-5 and XW-4, plants of *L. chinensis* and the local microbiota on the improvement of the saline-alkali soil property were summarized in Figure 17. Both the strains JL-5 and XW-4 provided GABA and proline for plants of *L. chinensis* to promote its growth and provided carbon sources and nutrients to soil microorganisms, which in turn alleviated soil salinization and improved the living environment of the strains JL-5 and XW-4. Furthermore, the well-established roots of *L. chinensis* could provide rhizosphere nutrients for soil microorganisms, ultimately enhancing the abundance of soil microbiota. Moreover, the soil microorganisms could promote the transformation of aspartate into Glu by the strain JL-5, promoting its effect on its saline-alkali tolerance and coordinating with XW-4 to provide carbon sources. Additionally, the acetate secreted by soil microorganisms could alleviate the saline-alkali stress on *L. chinensis*, ultimately promoting the growth of *L. chinensis*.

## 4. Materials and Methods

The experimental designs were based on the following factors: (1) the biochar produced by corn straw degradation not only improved the soil environment by providing more available carbon sources but also promoted the growth of *L. chinensis* and the proliferation of phosphate-solubilizing bacteria, e.g., the strain JL-5, to alleviate the soil salinization; (2) indicator plants of *L. chinensis* were cultivated in the saline-alkali soil mixed with corn straw and treated with the strains JL-5 and XW-4, and the physiological and biochemical characteristics of both *L. chinensis* and the soils were measured to evaluate the properties and the improvement of saline-alkali soils; and (3) the synergistic effects of the strains JL-5 and XW-4, the indicator plants of *L. chinensis* and the diversity and abundance of local microbiota on the improvement of saline-alkali soil environments were evaluated.

### 4.1. Isolation and Identification of Saline-alkali-Tolerant Bacteria

#### 4.1.1. Isolation and Screening of Saline-alkali-Tolerant Bacteria

The soil samples with a pH = 8.93, electrical conductance (EC) = 0.579 mS/cm and salinity level of 4.1 g/kg were collected from the saline-alkali soils in Zhenlai County (45.30 N and 123.05 E), Baicheng City, Jilin Province, China. The modified Luria–Bertani (LB) medium containing 2% NaCl, with pH adjusted using NaOH (1 mol L^−1^) to 9.0, was used for saline-alkali resistance screening, as previously described [76]. A 5 g soil sample was added to 50 mL of sterile water, shaken and mixed well to prepare the soil diluent and shaken again for 10 min. This process was repeated five times to fully dissolve the microorganisms in the sterile water. A total of 200 μL of the supernatant was added to the modified LB medium, incubated at 37 °C and 180 rpm for 24 h and then coated on the solid modified LB medium. Individual single bacterial colonies were selected for separation and purification. The isolated strains were coated in LB medium with 1% NaCl and pH values of 8.0, 9.0, 10.0 and 11.0, respectively, and in LB medium with a pH of 7 and NaCl values of 1.5%, 2.0%, 2.5% and 3.0%, respectively. The saline-alkali resistances of the isolated strains were evaluated as follows: (1) the isolated bacteria were cultured in LB medium with 1% NaCl and pH values of 8.0, 9.0, 10.0 and 11.0 adjusted using NaOH, respectively; and (2) the isolated bacteria were inoculated in LB medium with a pH of 7.0 and NaCl values of 1.5%, 2.0%, 2.5% and 3.0% respectively. The bacterial growth under these conditions was observed to determine the saline-alkali resistance of these strains. The strains with high saline-alkali resistances were selected for subsequent experiments.

#### 4.1.2. Functional Selection and Identification of Saline-alkali-Tolerant Bacteria

Previous studies showed that phosphate-solubilizing bacteria generated acidic substances to alleviate the high pH levels of saline-alkali soils [77]. In this study, the strains screened with strong saline-alkali resistance were coated on solid National Botanical Research Institute’s Phosphate (NBRIP) growth medium to further screen and purify saline-alkali-tolerant phosphate-solubilizing bacteria, which were inoculated onto phosphate-solubilizing fermentation medium and cultured at 37 °C and 180 rpm for 24 h. The supernatant was collected after ultrasonic treatment of the bacteria for 20 min and centrifuged at 12,000 *g* for 5 min. The phosphate-solubilizing capacity was determined by molybdenum antimony anti-colorimetry, with bacteria showing the highest phosphate-solubilizing capacity selected for 16S rRNA identification and subsequent experiments. DNA was extracted using the extraction kit from Tiangen Biotech Co., Ltd. (Beijing, China), following the manufacturer’s instructions. The primers 27F (5′-AGAGTTTTGATCATGGCTCAG-3′) and 1492R (5′-ACGGTTACCTTGTTACGACTT-3′) were used to amplify the 16S rRNA sequences using PCR. The comparative and phylogenetic analyses of 16S rRNA sequences were performed using DNAman 8.0 (https://www.lynnon.com/; accessed on 13 February 2023) and MEGA X (https://www.megasoftware.net/; accessed on 13 February 2023), respectively, to identify the saline-alkali-tolerant and phosphate-solubilizing bacterium as *Priestia aryabhattai* JL-5, with its 16S rRNA sequence deposited to the National Center for Biotechnology Information (NCBI; https://www.ncbi.nlm.nih.gov/; accessed on 13 February 2023; GenBank accession OQ443070). The neighbor-joining method in MEGA X with default parameters was used to construct the phylogenetic trees with bootstrap supports obtained based on 1000 replicates.

Studies have shown that the corn straw could increase the soil nutrients and soil microbial biomass [78]. Furthermore, cellulose-decomposing bacteria could degrade corn straw into carbon sources to improve soil nutrients for the growth of other microorganisms in the local microbiota [79]. In this study, the bacterial strains with strong saline-alkali tolerance were coated on solid carboxymethyl cellulose (CMC) medium to screen and purify the saline-alkali-resistant cellulose-degrading bacteria, which were inoculated onto the cellulase-producing medium and cultured at 37 °C and 180 rpm for 24 h. The cellulase-producing activity of the saline-alkali-resistant cellulose-decomposing bacteria was determined by the dinitrosalicylic acid (DNS) method, with bacteria showing the highest cellulase-producing ability retained for 16S rRNA identification and subsequent experiments. The comparative and phylogenetic analyses of 16S rRNA sequences were performed using DNAMAN 8.0 (https://www.lynnon.com/; accessed on 13 February 2023) and MEGA X (https://www.megasoftware.net/; accessed on 13 February 2023) to identify the saline-alkali-tolerant and cellulose-degrading bacterium as *Staphylococcus pseudoxylosus* XW-4, with its 16S rRNA sequence deposited to NCBI (https://www.ncbi.nlm.nih.gov/; accessed on 13 February 2023; GenBank accession OQ443074).

### 4.2. Transcriptome Sequencing and Analysis

#### 4.2.1. Bacterial Sample Preparation

A total of 10 mL of strain JL-5 culture solution was transferred to 100 mL modified LB culture medium and cultured at 37 ºC and 180 rpm for 24 h. Then, 10 mL of the bacterial solution was transferred to modified LB culture medium and cultured at 37 ºC and 180 rpm for 24 h. This process was repeated for five generations to obtain the strain JL-5 with stabilized functional inheritance. The control group was cultured in normal LB (pH = 7 and 1% NaCl) culture medium under the same conditions. The OD_600_ of the bacterial cultures under different saline-alkali conditions was measured. For transcriptome sequencing, the bacterial solutions of the control and the experimental groups were collected at 10 OD mL^−1^, centrifuged at 12,000 rpm for 10 min, and enriched, with the supernatant removed and the sample quickly frozen in liquid nitrogen for 5 min. The transcriptome sequencing of the bacterial samples was completed by Shanghai Meiji Biomedical Technology Co., Ltd. (Shanghai, China).

#### 4.2.2. Analysis and Annotation of Differentially Expressed Genes

Total RNA was extracted from bacterial samples by TRIzol (Invitrogen, Beijing, China), with the concentration and purity of the extracted RNA detected by a NanoDrop 2000 Spectrophotometer (Thermo Fisher Scientific Inc., Beijing, China). The integrity of RNA was detected by agarose gel electrophoresis to ensure a total amount of RNA of 2 μg, with a concentration larger than 100 ng/μL and an OD_260/280_ between 1.8 and 2.2. The Illumina Hiseq sequencing platform was used to obtain the 2 × 150 bp/300 bp reads by Tiangen Biotech Co., Ltd. (Beijing, China). The raw data of the transcriptome sequencing of bacterial samples were deposited to the NCBI database (accession PRJNA934267). The transcript expressions were calculated using the transcriptional reading mapped by Kalisto 0.43.1 (https://pachterlab.github.io/kallisto/; accessed on 12 December 2022), Salmon 0.8.2 (https://github.com/COMBINE-lab/salmon/; accessed on 12 December 2022) and RSEM 1.3.1. (http://deweylab.biostat.wisc.edu/rsem/; accessed on 12 December 2022) for each thousand bases per million fragments. Differential expression analysis was performed on the functional genes using DESeq2 1.24.0 (http://bioconductor.org/packages/stats/bioc/DESeq2/; accessed on 12 December 2022) and edgeR 3.24.3 (http://bioconductor.org/packages/stats/bioc/edgeR/; accessed on 12 December 2022) based on |log_2_(fold change)| > 1 and P < 0.05 to evaluate the difference between two high-quality samples and to identify the differentially expressed genes (DEGs). The DEGs were further annotated using the Gene Ontology (GO) database (www.geneontology.org/; accessed on 12 January 2023), BLAST+ 2.3.0 (https://ftp.ncbi.nlm.nih.gov/blast/executables/blast+/2.3.0/; accessed on 12 January 2023) and Diamond 0.8.35 (https://github.com/bbuchfink/diamond/; accessed on 12 January 2023). The statistical analysis of GO annotation was performed using Goatools (https://github.com/tanghaibao/goatools/; accessed on 12 January 2023). The metabolic pathway enrichment analysis of DEGs was performed using the Kyoto Encyclopedia of Genes and Genomes (KEGG) database (www.genome.jp/kegg/; accessed on 12 January 2023) and the Fisher exact test of the online platform of Meiji Biology (https://www.majorbio.com/; accessed on 12 January 2023).

#### 4.2.3. Determination of Biochemical, Physiological and Morphological Characteristics of Bacteria

The strain JL-5 (1 mL with OD_600_ 0.8) was inoculated into 100 mL normal LB medium (pH = 7 and 1% NaCl) and modified LB medium (pH = 8 and 1.5% NaCl; pH = 9 and 2.0% NaCl, with pH levels adjusted using NaOH), respectively, and cultured at 37 °C and 180 rpm for 1 d. Then, the fermentation broth was collected and treated with the ultrasonic cell breaker (SCIENTZ JY92-IIN, Scientz Biotechnology Co., Ltd., Ningbo, China) at 200 W for 40 min to break the bacterial cells and centrifuged at 12,000 rpm for 2 min, with the supernatant collected to measure the contents of lactic acid, glutamine synthetase (GS) and proline. The bacterial solutions were dried and used for the morphological observations of the saline-alkali-tolerant and phosphate-solubilizing bacteria under normal and saline-alkali conditions using scanning electron microscopy (SEM; Hitachi SU8010, Hitachi Limited Co., Ltd., Tokyo, Japan).

### 4.3. Promoted Growth and Alleviated Saline-alkali Stress of Leymus chinensis by the Strains JL-5 and XW-4

Both the strains JL-5 and XW-4 were inoculated in LB medium and cultured at 37 ºC and 180 rpm for 2 d. Then, the OD_600_ values of both strains were determined to identify the bacterial solutions of both strains with identical OD_600_ values, each containing viable bacterial cells > 2 × 10^8^ mL^−1^. A total of 10 mL of the mixed bacterial solution of the strains JL-5 and XW-4 at the ratios of 1:1, 1:2, 2:1, 1:3, 3:1, 2:3 and 3:2, respectively, was evenly applied to 1000 g of soil, which was used to randomly plant 50 seeds of *L. chinensis* in each pot. Based on the germination rate of *L. chinensis* seeds, the optimal ratio (2:1) of the two strains in the bacterial solution was determined and used in the following experiments. The seeds of *L. chinensis* were grown in soils treated without a bacterial solution (control), with a bacterial solution of either one of the two strains of JL-5 and XW-4 and with a bacterial solution of both strains, respectively, for 15 d. The physiological and biochemical variations in plants of *L. chinensis* at the tillering stage under different treatments were evaluated. Fresh leaves were collected, immediately treated with liquid nitrogen and kept at −80 °C for further experiments. In order to evaluate the growth-promoting effects and stress-alleviating effects of bacterial solutions on plants of *L. chinensis*, the contents of SOD, POD, CAT, proline and MDA were determined by the azo-blue tetrazole method, the guaiacol method, the ultraviolet absorption method, the ninhydrin method and the thiobarbituric acid (TBA) method, respectively. Each experiment was repeated with three biological replicates.

### 4.4. Evaluation of Soil Properties and Microbiota

A total of 50 seeds of *L. chinensis* were sown in each of six plastic pots with soils treated with a bacterial solution of the strains JL-5 and XW-4 of the optimal ratio identified above for 15 d until tillering. The soils of six control groups were not treated with bacterial solutions. Soil samples were randomly selected from a total of six different positions in each of the six pots and stored at −80 °C for the further determination of the contents of available potassium (K), phosphorus (P) and sodium (N) and organic matter using the flame photometric method, the 0.5 mol L^−1^ NaHCO3 method, the alkali diffusion method and the potassium dichromate volumetric method, respectively. Then, the 16S rRNA amplicon sequencing technology was used to investigate the species diversity, richness, and relationship network based on both QIIME2 2019.4 (https://qiime2.org/; accessed on 15 December 2022) and Vsearch (https://github.com/torognes/vsearch/; accessed on 15 December 2022) to cluster the operational taxonomic units (OTUs) based on high-quality sequencing data. Both alpha and beta diversities of each sample were investigated based on the abundance distribution of OTUs. The functional enrichment of the soil microbiota was performed based on the MetaCyc Metabolic Pathway Database (http://metacyc.org/; accessed on 15 December 2022). Then, the SparCC (https://github.com/dlegor/Sparcc/; accessed on 15 December 2022) were applied to build the association network of OTUs based on the composition and distribution of taxa in each sample. The igraph (https://igraph.org/; accessed on 15 December 2022) was used to calculate the topological index, to identify the dominant taxa and to construct the association network. Finally, the microbial metabolic functions of the soil samples were predicted using PICRUSt2 (https://github.com/picrust/picrust2/; accessed on 15 December 2022) and HUMAnN2 (http://huttenhower.sph.harvard.edu/humann2/; accessed on 15 December 2022) based on the 16S rRNA sequencing, with the differential pathways and their compositions identified. The sequencing and taxonomic annotations were performed by Nanjing Personal Biotechnology Co., Ltd. (Nanjing, China).

### 4.5. Statistical Analysis

SPSS Statistics 26 was used to analyze the data, calculate the standard deviation of each experimental group, and perform the one-way ANOVA. The significant differences were determined based on *p* < 0.05 using the least significance difference (LSD) analysis. GraphPad Prism 9 was used to generate the graphs with statistical analysis.

## 5. Conclusions

In conclusion, our results showed that it was more efficient to apply the combined biological strategies to deal with soil salinization in order to achieve the synergistic effects on the improvement of saline-alkali soils. Our study revealed a distinctive molecular mechanism of the strain JL-5 in response to saline-alkali stress. In particular, with the treatment of bacterial solutions containing both the strains JL-5 and XW-4, more carbon sources were provided by the degradation of corn straw by the strain XW-4, while the strain JL-5 could initiate a series of molecular responses to maintain its physiological and biochemical activities to improve its saline-alkali tolerance, e.g., the strain JL-5 initially responded to the saline-alkali stress via glutamate metabolism (enhanced via the aspartic acid pathway). A large number of asymmetric septations were required to maintain the replication and apoptosis, while many significantly enriched metabolic pathways were involved in the apoptosis and replication in the strain JL-5 under saline-alkali stress, suggesting that the maintenance of asymmetric cell division was closely related to glutamate metabolism. Moreover, the strain JL-5 enhanced its saline-alkali tolerance through the interactions with other bacteria in the soil microbiota to enrich the microbial community in the soil. The strain JL-5 further improved the saline-alkali soil property by providing proline to the indicator plants of *L. chinensis* to enhance the activities of antioxidant enzymes in these plants. Together with the carbon sources produced by corn straw, which was degraded by the strain XW-4, the strain JL-5 ultimately promoted the vegetative growth of *L. chinensis*. The application of corn straw improved the mechanical strength of saline-alkali soil, making the soil loose and conducive to the growth of roots of *L. chinensis*. This study has provided strong experimental evidence to support the application of the multi-biological interactions among several organisms in saline-alkali land improvement due to their advantages in response to saline-alkali stress with both biochemical and physiological changes. Our study provides a new direction for future investigations into the bacterial response to saline-alkali stress and strong experimental evidence to support the application and commercial development of combined biological strategies in the improvement of saline-alkali soil environments.

## Figures and Tables

**Table 1:** Characteristics of transcriptome sequencing and assembly of *Priestia aryabhattai* JL-5 cultured under control (pH = 7.0 and 1% NaCl; JL-5-7) and saline-alkali conditions (pH = 9.0 and 2% NaCl; JL-5-9) based on the reference genome of *Bacillus cereus* (assembly ASM222028v1 of GenBank accession GCF_002220285.1).

Characteristics	JL-5-7 (%)	JL-5-9 (%)
Total raw read	30,741,042	29,035,034
Genome mapped read (%)	28,333,104 (92.2)	26,955,132 (92.8)
Unmapped read (%)	2,407,938 (7.8)	2,079,902 (7.2)
Uniquely mapped read (%)	26,264,786 (85.4)	25,249,848 (87.0)
Coding sequence (CDS) mapped reads (%)	25,504,272 (88.2)	24,246,954 (88.2)

**Table 2:** Enrichment analysis based on the Kyoto Encyclopedia of Genes and Genomes (KEGG) database of a total of 10 differentially expressed genes (6 up-regulated and 4 down-regulated) involved in glutamate metabolism and identified in *Priestia aryabhattai* JL-5 under the saline-alkali conditions. Symbols “↑” and “↓” indicate up-regulation and down-regulation, respectively. Enzymes with expression levels regulated are given in the pathways that they are involved in. GS, glutamine synthetase; GluL, glutamate-ammonia ligase; Hsp90, heat shock protein 90; RIPK3, receptor-interacting protein kinase 3; LepA, leader peptide A; SdhA, succinate dehydrogenase A; HTPB, high temperature protein B; ENO1, enolase 1; GAPDH, glyceraldehyde-3-phosphate dehydrogenase; PFKL, phosphofructokinase of liver type; PGK1, phosphoglycerate kinase 1; LDHA, lactate dehydrogenase A; HIF-1, hypoxia-inducible factor 1.

Gene ID [log2fc(JL-5-9/JL-5-7)]	Gene: Description	KEGG Pathway: Description	Pathway Component
FORC47_RS19095 [2.724 ↑]	*glnA*: type I glutamate-ammonia ligase	K01915: glutamine synthetase	map00630; map00220; map00910; map02020; map00250; map04727 (GS); map04724; map04217 (GluL, Hsp90 and RIPK3)
FORC47_RS26675 [2.343 ↑]	*ftsX*: permease-like cell division protein FtsX	K09811: cell division transport system permease protein	map02010
FORC47_RS20430 [1.987 ↑]	*FORC47_RS20430*: protein-glutamine γ-glutamyltransferase	K00686: protein-glutamine gamma-glutamyltransferase	–
FORC47_RS01880 [1.551 ↑]	*gatA*: Asp-tRNA(Asn)/Glu-tRNA(Gln) amidotransferase subunit GatA	K02433: aspartyl-tRNA(Asn)/glutamyl-tRNA(Gln) amidotransferase subunit A	map00970
FORC47_RS01875 [1.389 ↑]	*gatC*: Asp-tRNA(Asn)/Glu-tRNA(Gln) amidotransferase subunit GatC	K02435: aspartyl-tRNA(Asn)/glutamyl-tRNA(Gln) amidotransferase subunit C	map00970
FORC47_RS01885 [1.054 ↑]	*gatB*: Asp-tRNA(Asn)/Glu-tRNA(Gln) amidotransferase subunit GatB	K02434: aspartyl-tRNA(Asn)/glutamyl-tRNA(Gln) amidotransferase subunit B	map00970
FORC47_RS04480 [−1.028 ↓]	*FORC47_RS04480*: type 1 glutamine amidotransferase	–	–
FORC47_RS01550 [−1.150 ↓]	*groL*: chaperonin GroEL	K04077: chaperonin GroEL	map03018; map05152; map04212; map05134 (LepA, SdhA and HTPB); map04940
FORC47_RS26415 [−1.648 ↓]	*eno:* phosphopyruvate hydratase	K01689: enolase	map00680; map00010; map03018; map04066 (ENO1, GAPDH, PFKL, PGK1, LDHA and HIF-1)
FORC47_RS03370 [−4.241 ↓]	*glnH*: glutamine ABC transporter substrate-binding protein GlnH	K10039: aspartate/glutamate/glutamine transport system substrate-binding protein	map02010

**Table 3:** Measurements of aboveground and underground portions of *Leymus chinensis* plants grown in control (without bacterial solution) and separate and combined treatments of *Priestia aryabhatai* JL-5 and *Staphylococcus pseudoxylosus* XW-4 for 14 d. Data are presented as the mean ± standard deviation (SD). Different superscript letters, a, b and c, represent the significant difference based on *p* < 0.05 in each column.

Treatment	Aboveground Portion (cm)	Underground Portion (cm)	Plant at Tillering Stage (%)
Control	5.3 ± 0.7^c^	3.5 ± 0.5^c^	22 ± 6^c^
JL-5	5.4 ± 0.4^c^	6.1 ± 0.8^b^	78 ± 4^a^
XW-4	5.6 ± 0.6^b^	4.1 ± 0.4^c^	51 ± 5^b^
JL-5 and XW-4	7.4 ± 0.6^a^	6.6 ± 1.0^a^	57 ± 3^b^

## Data Availability

The raw data of the transcriptome sequencing of bacterial samples of *Priestia aryabhattai* JL-5 were deposited to the NCBI database (https://www.ncbi.nlm.nih.gov/bioproject/PRJNA934267/; accessed on 13 February 2023). The 16S rRNA sequences of *Priestia aryabhattai* JL-5 and *Staphylococcus pseudoxylosus* XW-4 were deposited to the NCBI database (https://www.ncbi.nlm.nih.gov/nuccore/OQ443074.1/; https://www.ncbi.nlm.nih.gov/nuccore/OQ443070.1/; accessed on 13 February 2023).

## Supplementary Materials

The following supporting information can be downloaded at: https://www.mdpi.com/article/10.3390/ijms24097737/s1.
