# Supplementary material for: Saline-Alkali Soil Property Improved by the Synergistic Effects of Priestia aryabhattai JL-5, Staphylococcus pseudoxylosus XW-4, Leymus chinensis and Soil Microbiota
Source: Int J Mol Sci. 2023 Apr 23;24(9):7737. doi: 10.3390/ijms24097737 (PMC10178608; doi:10.3390/ijms24097737)
Supplement: Supplementary file 1 [file ijms-24-07737-s001.zip › FIgure S2.pdf]

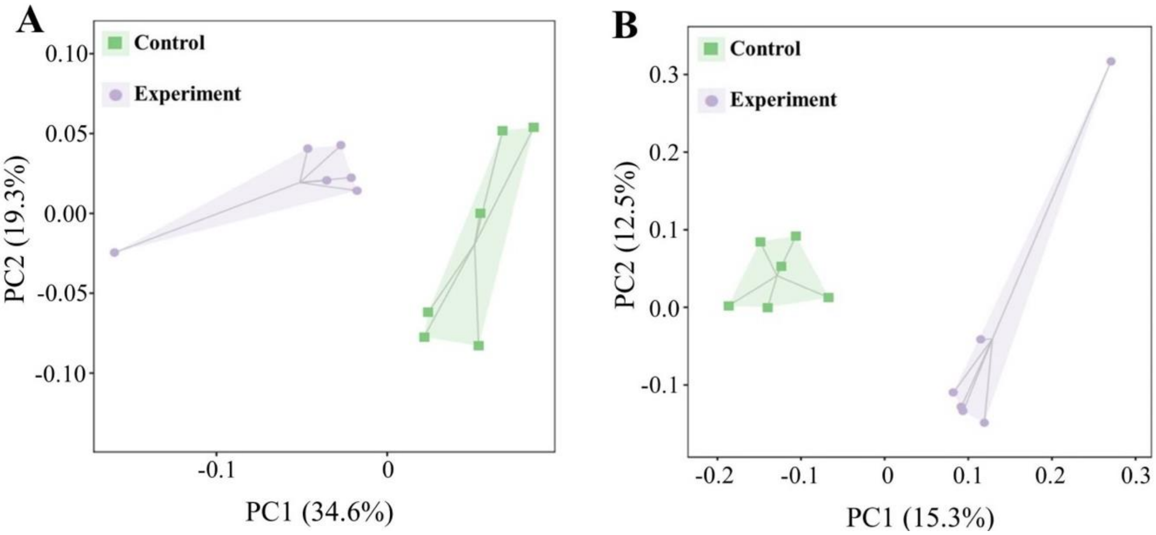

Figure S2. Principal coordinate analyses of the soil samples of 6 control (without bacterial treatment) and 6 experimental (treated with bacterial mixture of *Priestia aryabhattai* JL-5 and *Staphylococcus pseudoxylosus* XW-4 with the ratio of 2:1) groups based on Bray-Curtis distance (**A**) and weighted UniFrac distance (**B**).
