# Supplementary material for: Saline-Alkali Soil Property Improved by the Synergistic Effects of Priestia aryabhattai JL-5, Staphylococcus pseudoxylosus XW-4, Leymus chinensis and Soil Microbiota
Source: Int J Mol Sci. 2023 Apr 23;24(9):7737. doi: 10.3390/ijms24097737 (PMC10178608; doi:10.3390/ijms24097737)
Supplement: Supplementary file 1 [file ijms-24-07737-s001.zip › Figure S3.pdf]

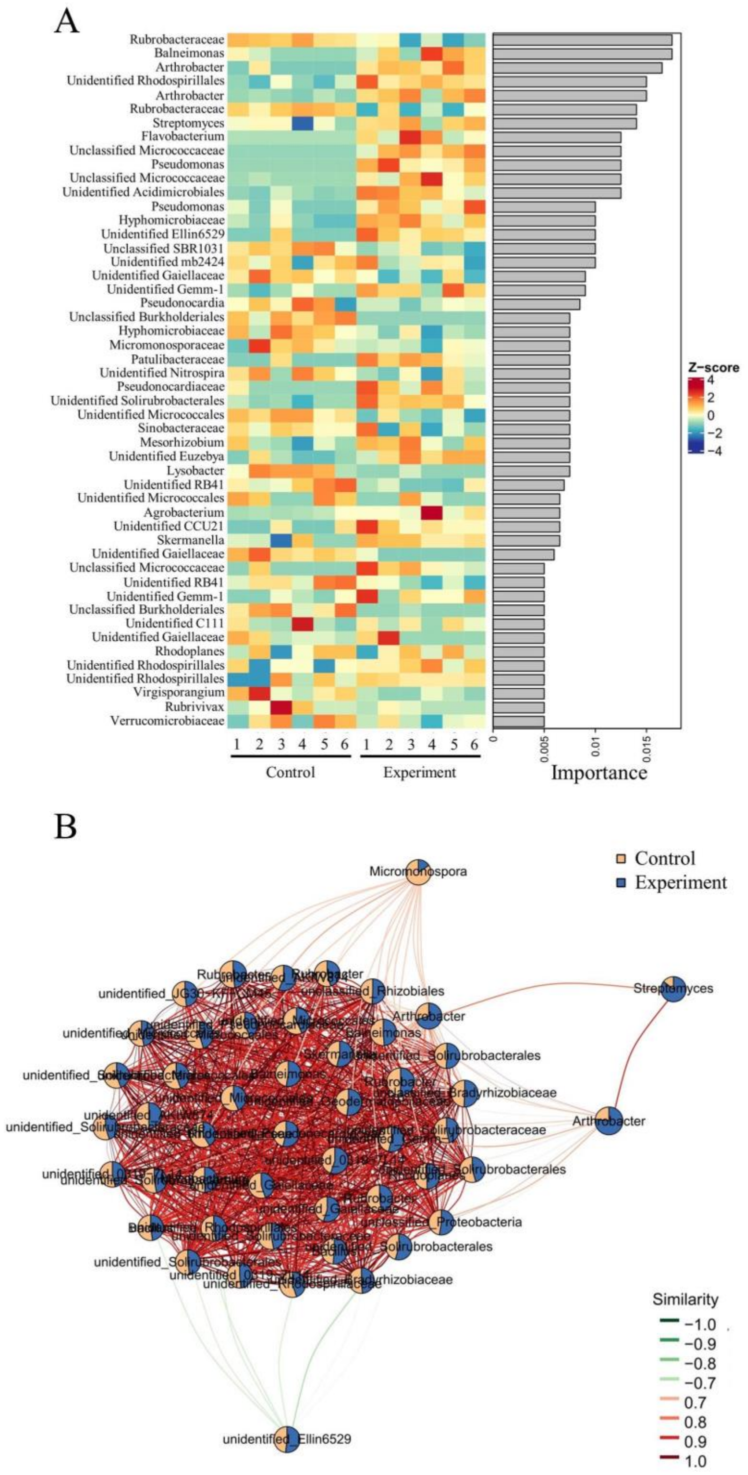

Figure S3. Effects of combined bacterial solutions of both *Priestia aryabhattai* JL-5 and *Staphylococcus pseudoxylosus* XW-4 on the relationships among soil microorganisms. (**A**) Heatmap of top 50 operational taxonomic units (OTUs) with the significant differences between the experimental and control groups. (**B**) Network of relatively dominant taxa with the taxonomic abundance indicated in the pie graphs.
