# Supplementary material for: Saline-Alkali Soil Property Improved by the Synergistic Effects of Priestia aryabhattai JL-5, Staphylococcus pseudoxylosus XW-4, Leymus chinensis and Soil Microbiota
Source: Int J Mol Sci. 2023 Apr 23;24(9):7737. doi: 10.3390/ijms24097737 (PMC10178608; doi:10.3390/ijms24097737)
Supplement: Supplementary file 1 [file ijms-24-07737-s001.zip › Table S2.pdf]

Table S2. Alpha diversity analysis of the microbial community in the soils of 6 control groups treated with no bacterial solutions and 6 experimental groups treated with mixed bacterial solutions of both *Priestia aryabhattai* JL-5 and *Staphylococcus pseudoxylosus* XW-4 (with the ratio of 2:1).

**Sample**	**Chao1**	**Simpson**	**Shannon**	**Observed species**	**Good's coverage**
Control group					
Control 1	6191	0.998	11.088	5733	0.976
Control 2	6565	0.998	11.171	5936	0.972
Control 3	6431	0.998	11.117	5774	0.973
Control 4	5689	0.998	10.966	5295	0.979
Control 5	6173	0.998	11.049	5610	0.975
Control 6	6537	0.996	10.811	5646	0.970
Experimental group					
Experiment 1	7016	0.998	11.160	6090	0.968
Experiment 2	6282	0.998	11.046	5714	0.974
Experiment 3	6242	0.998	10.946	5568	0.973
Experiment 4	6937	0.998	11.040	6173	0.969
Experiment 5	6948	0.998	11.213	6428	0.972
Experiment 6	7052	0.998	11.154	6401	0.970
